# Mechanical Performance of Compacted Cement-Stabilized Loess: A Porosity-Based Multi-Property Modeling Framework

**DOI:** 10.3390/ma19214487

**Published:** 2026-10-24

**Authors:** Jian Guo, Qigang Chan, Changjiang Dong, Chunfa Liu, Ziyu Lu, Lin Li, Kai Yao

**Affiliations:** 1 School of Qilu Transportation, Shandong University, Jinan 250002, China; guojian935@sdu.edu.cn (J.G.); 202535637@mail.sdu.edu.cn (Z.L.); 202415463@mail.sdu.edu.cn (L.L.); 2 Jining Hongxiang Highway Survey Design & Research Institute Co., Ltd., Jining 272000, China; chanqigang@gmail.com (Q.C.); dongchangjiang88@gmail.com (C.D.); liuchunfa819@gmail.com (C.L.); 3 School of Civil Engineering, Northwest Minzu University, Lanzhou 730124, China; 4 Xinjiang Research Institute of Shandong University, Changji 831100, China

**Keywords:** cement-stabilized loess, porosity, mechanical properties, curing behavior, predictive modeling

## Abstract

Compacted cement-stabilized loess is widely used in civil infrastructure, yet existing strength models are often based on water-related variables and are difficult to transfer across different mechanical properties of unsaturated loess. This study develops a porosity-based modeling framework linking cement dosage, initial void state, and curing time to the mechanical response of compacted cement-stabilized loess. Three representative strength models were reformulated using cement volumetric content, void volumetric content, and their derived variables. Unconfined compressive strength (UCS), triaxial shear, California bearing ratio (CBR), static resilient modulus (M_R_), and uniaxial consolidation tests were used to assess the formulations. The dual-power volumetric model provided the most consistent multi-property representation by separately accounting for cement volume and void volume. A normalized hyperbolic function effectively described curing-time evolution and was more physically consistent with long-term stabilization than the logarithmic form. *UCS*-derived parameters were directly transferable to cohesion (*c*), whereas internal friction angle (*φ*) and *M*_R_ required recalibration or baseline correction; *CBR* transferability was promising under fixed standard compaction. A literature-based generalized hyperbolic relationship further captured normalized *UCS* development across different loess sources. The proposed framework links material composition, compacted void state, and curing history to mechanical performance, providing a basis for performance-oriented assessment of cement-stabilized loess. The present results are based primarily on laboratory-prepared specimens, and further validation using independent field data is required before broader engineering application.

## 1. Introduction

Loess is a typical problematic geomaterial widely distributed in arid and semi-arid regions and frequently encountered in transportation infrastructure, slope engineering, and shallow foundations [1]. Due to its metastable structure, high porosity, weak cementation, and strong water sensitivity, loess is prone to wetting-induced collapse, strength degradation, and structural instability [2,3]. These characteristics pose significant challenges to the long-term performance and safety of geotechnical structures. Accordingly, improving the engineering properties of loess and establishing reliable evaluation methods remain important topics in geotechnical engineering.

The improvement of collapsible loess primarily involves a combination of physical and chemical methods [4,5]. In recent years, chemical stabilization using industrial solid wastes has attracted increasing attention as a sustainable alternative to cement [6,7]. However, its large-scale engineering application remains limited due to three major constraints: (i) the heterogeneous sources and fluctuating compositions of solid wastes, along with the lack of unified quality control standards; (ii) lack of an integrated and scalable production system; and (iii) potential environmental risks associated with pollutant leaching [8,9]. In contrast, cement stabilization remains a widely used and well-established technique due to its rapid strength gain, construction simplicity, and well-established engineering practice [10]. Extensive experimental and engineering studies have demonstrated the effectiveness of cement in improving the strength and deformation resistance of loess. As a result, a considerable amount of data on the engineering behavior of cement-stabilized loess has been accumulated.

Despite these advances, the current understanding and application of cement-stabilized loess still face several limitations. In practical engineering, key design parameters such as cement dosage are often determined largely on the basis of experience, and the long-term performance of stabilized loess is rarely predicted in a systematic manner. More importantly, although many studies have examined the influence of cement content, density, and curing age on the mechanical properties of stabilized soils, a unified and physically interpretable framework suitable for cement-stabilized unsaturated loess is still lacking. This limitation restricts both rational design and reliable quality control in engineering practice.

Previous studies have identified several key factors controlling the engineering properties of stabilized soils, including binder type, binder content, water content, curing period, and curing conditions [11,12,13,14]. To quantify these effects, a variety of strength evaluation models have been proposed (Table 1). Most of these models, however, were established for saturated or near-saturated fine-grained soils and rely heavily on water-related variables such as water content or water–cement ratio. Such formulations are generally effective for stabilized soft clays, dredged soils, and other saturated geomaterials, where the relationship between water content and internal void structure is relatively stable. By contrast, loess is typically an unsaturated soil, and there is no necessary one-to-one correspondence between water content and porosity. As a result, directly applying conventional water-based models to cement-stabilized loess may lack physical rationality and predictive robustness.

In addition to this limitation, many existing models are formulated only for a single mechanical index, most commonly *UCS*, without clearly linking the predicted property to specific engineering applications. In practice, different engineering fields require different evaluation indices. For example, *UCS* is commonly used in the quality control of road base and subbase materials, *CBR* and *M*_R_ are important indicators for subgrade evaluation, and shear strength parameters are essential for slope stability analysis [25,26,27,28]. Therefore, evaluation methods intended for engineering application should not only account for the combined effects of controllable engineering factors (e.g., cement dosage, compactness, and curing time), but also provide a practical bridge among different engineering indices. Furthermore, many existing models involve complicated forms and numerous fitting parameters, which reduces their universality and limits their applicability in engineering design and field quality assessment.

Against this background, this study aims to establish a porosity-based modeling framework for the mechanical performance of compacted cement-stabilized loess. Three representative strength models were reformulated using cement volumetric content and initial void volumetric content to represent the contributions of cementitious bonding and compacted pore structure. A normalized hyperbolic function was subsequently introduced to describe curing-time evolution. Based on laboratory tests, including UCS, triaxial shear, CBR, M_R_, and uniaxial consolidation tests, the effects of cement dosage, compactness, and curing time on *UCS*, shear strength parameters, and compressibility were systematically quantified. For *CBR* and *M*_R_, the applicability of the proposed formulations was mainly examined with respect to cement dosage and curing time under a fixed standard compaction condition. Furthermore, simplified relationships between *UCS* and other engineering indices were established to enable rapid estimation in practical applications. Finally, a generalized hyperbolic expression based on literature data from different regions was developed to further improve model universality. The proposed framework is expected to provide both theoretical support and practical guidance for the design, construction, and quality prediction of cement-stabilized loess used in road bases, subgrades, slopes, and foundations.

## 2. Porosity-Based Modeling Framework

The methodology of this study was developed to transform existing strength-oriented models into a unified evaluation framework for multiple engineering indices of cement-stabilized loess. The framework is based on the premise that the engineering response of stabilized loess is governed by two coupled mechanisms: cementation development and skeleton densification. Cementation development represents the bonding and filling effects generated by cement hydration, whereas skeleton densification reflects the particle contact condition, void distribution, and load-transfer capacity of the compacted loess matrix [29,30,31,32]. Under a prescribed curing environment, these mechanisms can be primarily characterized by cement dosage, compactness, and curing time. More importantly, these three variables are not only mechanistically relevant to the engineering response of stabilized loess but also practically controllable during mixture design, field compaction, and curing management.

Based on this concept, Models (5), (6), and (11) in Table 1 were selected as the original formulations and reformulated into Equations (12)–(14), respectively. Specifically, Equation (12) retains the inverse power-law structure of Model (5), replacing the water-to-cement ratio with the void-to-cement volumetric ratio (*V*_V_/*C*_V_). Equation (13) preserves the cement-content threshold and solid-volume term (*Y*^N^) of Model (6), while expressing cement dosage as the mass fraction of cement in the mixture (C*). Equation (14) adopts the dual-power structure of Model (11), replacing the cement and water-related variables with cement volumetric content (*C*_V_) and void volumetric content (*V*_V_), respectively. The curing-time effect is considered separately through Equations (19) and (20). These models were adopted because they are mathematically concise, involve a limited number of fitting parameters, and are capable of embedding the effects of cement dosage and compactness into physically interpretable variables. In these formulations, the modified parameters are intended to represent the relative roles of cementitious bonding and void-structure characteristics in controlling the engineering response of stabilized loess. Nevertheless, the three formulations differ in mathematical structure, and their predictive reliability and parameter transferability cannot be assumed a priori. Their applicability must therefore be evaluated against experimental results, with the ultimate objective of identifying a practical model form that combines prediction accuracy, parameter interpretability, and engineering usability.

(12) $$EI=\frac{a}{{\left(V_{V}/C_{V}\right)}^{x}}+b$$

(13) $$EI=K_{1}\left(C^{*}-C_{0}^{*}\right)Y^{N}$$

(14) $$EI=K_{2}\frac{C_{V}^{m}}{V_{V}^{n}}$$

In the modified models, *EI* denotes a generalized engineering index, including *UCS*, shear strength parameters (*c* and *φ*), *CBR*, *M*_R_, and *a*_1-2_ (the coefficient of compressibility determined over the vertical stress interval of 100–200 kPa in the uniaxial consolidation test, MPa^−1^). The parameters a, b, x, K_1_, N, K_2_, m, and n are fitting parameters, among which K_1_ and K_2_ are associated with curing age. To establish dimensionally consistent and physically interpretable variables, cement dosage and void state are expressed by cement volumetric content (*C*_V_) and void volumetric content (*V*_V_), respectively, as defined in Equations (15) and (16). The parameter C* represents the mass fraction of cement in the cement–loess mixture, while *C*_0_* denotes the threshold cement content required to initiate effective strength development, as given in Equation (17). The parameter *Y*, calculated using Equation (18), is introduced to characterize the solid-volume contribution and the influence of void distribution within the stabilized specimen.

The three modified models differ in how they describe the interaction between cement dosage and void structure. In Equation (12), the engineering index is correlated with a compound volumetric variable involving *V*_V_ and *C*_V_, which provides a simple expression of the balance between pore volume and cement volume. In Equation (13), the cementation effect is described by the cement content exceeding a threshold value, while the compactness effect is introduced through the term *Y*^N^. This formulation enables the threshold effect of cement addition and the sensitivity to void distribution to be evaluated separately. In Equation (14), *C*_V_ and *V*_V_ are assigned independent power exponents, allowing the cementation and void-structure effects to vary non-proportionally. This form is therefore expected to provide greater flexibility when different engineering indices respond differently to cement dosage and compactness.

(15) $$C_{V}=\frac{100\left(m_{\mathrm{cement}}/\rho_{s,\;\mathrm{cement}}\right)}{V_{S}}$$

(16) $$V_{V}=100-\frac{100\left(m_{\mathrm{cement}}/\rho_{s,\;\mathrm{cement}}+m_{\mathrm{loess}}/\rho_{s,\;\mathrm{loess}}\right)}{V_{S}}$$

(17) $$C^{*}=\frac{m_{\mathrm{cement}}}{m_{\mathrm{loess}}+m_{\mathrm{cement}}}\times 100$$

(18) $$Y=\frac{V_{\mathrm{cement}}+V_{\mathrm{loess}}}{V_{S}}\times 100$$

In Equations (15)–(18), *m*_cement_ and *m*_loess_ refer to the initial dry masses of cement and loess (g) used in specimen preparation, respectively, *ρ*_s,cement_ and *ρ*_s,loess_ denote the particle densities of the initial raw materials (g/cm^3^), respectively, and *V*_cement_, *V*_loess_, and *V*_S_ refer to the cement volume, loess volume and specimen volume (cm^3^) immediately after compaction, respectively.

**Figure 1:**
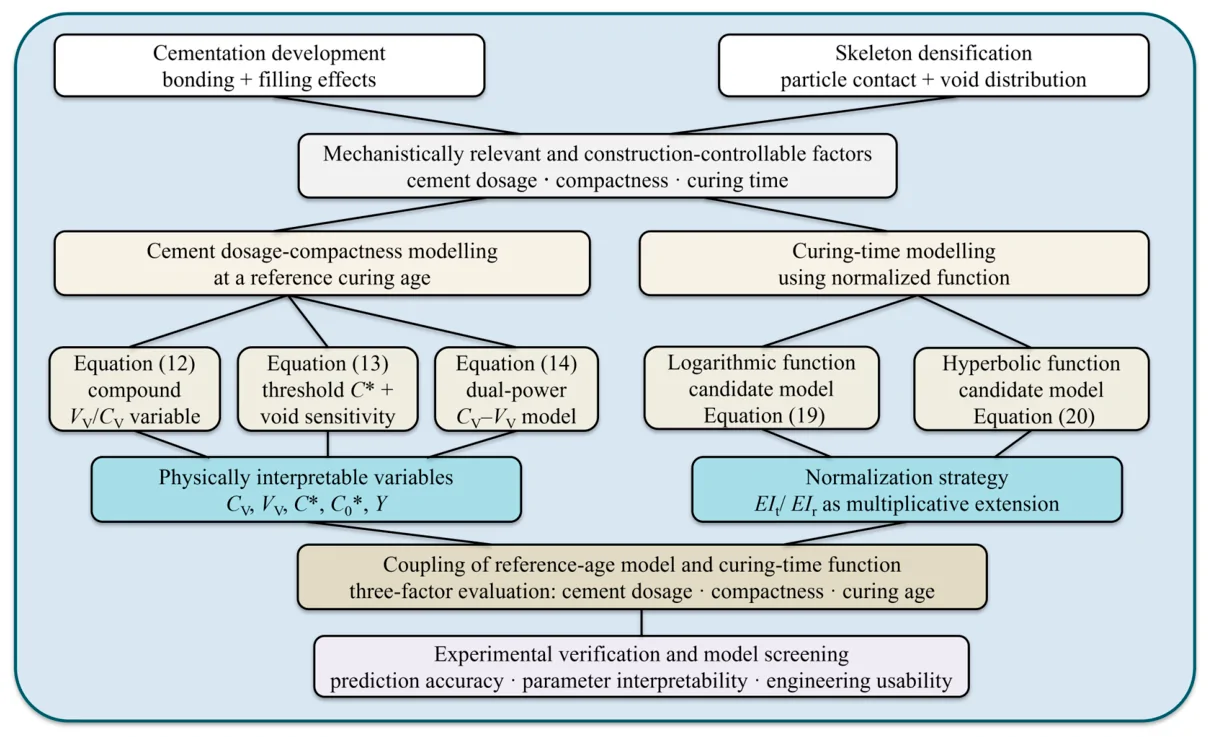
Methodological roadmap for the evaluation framework.

In previous studies on stabilized soils, the influence of curing time on engineering properties has commonly been characterized using logarithmic and hyperbolic functions, as reflected by several representative models summarized in Table 1. Therefore, these two types of functions were selected as candidate formulations for describing the curing-time-dependent evolution of engineering indices, with their suitability to be evaluated against the experimental data in Section 4.1. To further couple the curing-time effect with the cement dosage–compactness models established at a reference curing age, a normalization strategy was introduced following the approach proposed by Yao et al. [33]. Specifically, each engineering index at curing age *T* was normalized by its corresponding reference value at curing age *T*_0_, and the logarithmic and hyperbolic functions were reformulated in terms of the normalized engineering index *EI*_t_/*EI*_r_, as expressed in Equations (19) and (20). This treatment enables the curing-time function to act as a multiplicative extension of the reference-age model. In addition, normalization converts the curing-time effect into a dimensionless form, reducing the dependence of the time-dependent function on the magnitude and physical unit of a specific engineering index and improving its applicability to different engineering properties. The applicability of the two normalized functions to cement-stabilized loess is evaluated in Section 4, and the more suitable function is subsequently used to establish the three-factor evaluation models. The overall methodological procedure, including model reformulation, curing-time normalization, and three-factor model development, is schematically illustrated in Figure 1.

(19) $$\frac{EI_{t}}{EI_{r}}=A\ln T+B$$

(20) $$\frac{EI_{t}}{EI_{r}}=\frac{\alpha {\left(T/T_{0}\right)}^{\gamma }}{1+\beta {\left(T/T_{0}\right)}^{\gamma }}$$

## 3. Materials and Experimental Methods

### 3.1. Materials

The loess used in this study was collected from an excavation site at a depth of 6–10 m in Lanzhou, Gansu Province, China. The sampled loess was air-dried, crushed, and passed through a 0.5 mm sieve before specimen preparation. Its basic physical and engineering properties are summarized in Table 2. The *c* and *φ* reported in Table 2 were determined through triaxial compression tests on untreated loess specimens compacted at their maximum dry density (*MDD*) and optimum water content (*OWC*). Ordinary Portland cement (P.O 42.5), supplied by Gansu Qilianshan Cement Group Co., Ltd. (Lanzhou, China), was used as the stabilizing agent, with a specific gravity of 3.25 provided by the manufacturer.

### 3.2. Specimen Preparation

The cement dosage was determined by considering the typical requirements of different engineering applications in collapsible loess regions. According to relevant Chinese specifications for highway subgrades and building construction in loess areas [34,35,36], cement dosages of approximately 4% are commonly used for subgrade, slope, and foundation improvement, whereas higher dosages of approximately 10% may be required for road base and subbase materials. Therefore, cement contents of 2%, 4%, 6%, 8%, and 10% were selected in this study, covering both low-dosage improvement and relatively high-dosage stabilization scenarios.

The *MDD* and *OWC* of each cement–loess mixture were determined through standard compaction tests following JTG 3430-2020 [37], as listed in Table 3. Before cement addition, the required amount of water was first mixed with the air-dried loess until a uniform moisture condition was achieved. The wetted loess was then sealed in plastic bags for 24 h to ensure moisture redistribution. Cement was subsequently added and mixed thoroughly with the pre-wetted loess. To reduce the potential strength loss associated with premature cement hydration before compaction, all specimens were compacted within 1 h after cement addition.

**Figure 2:**
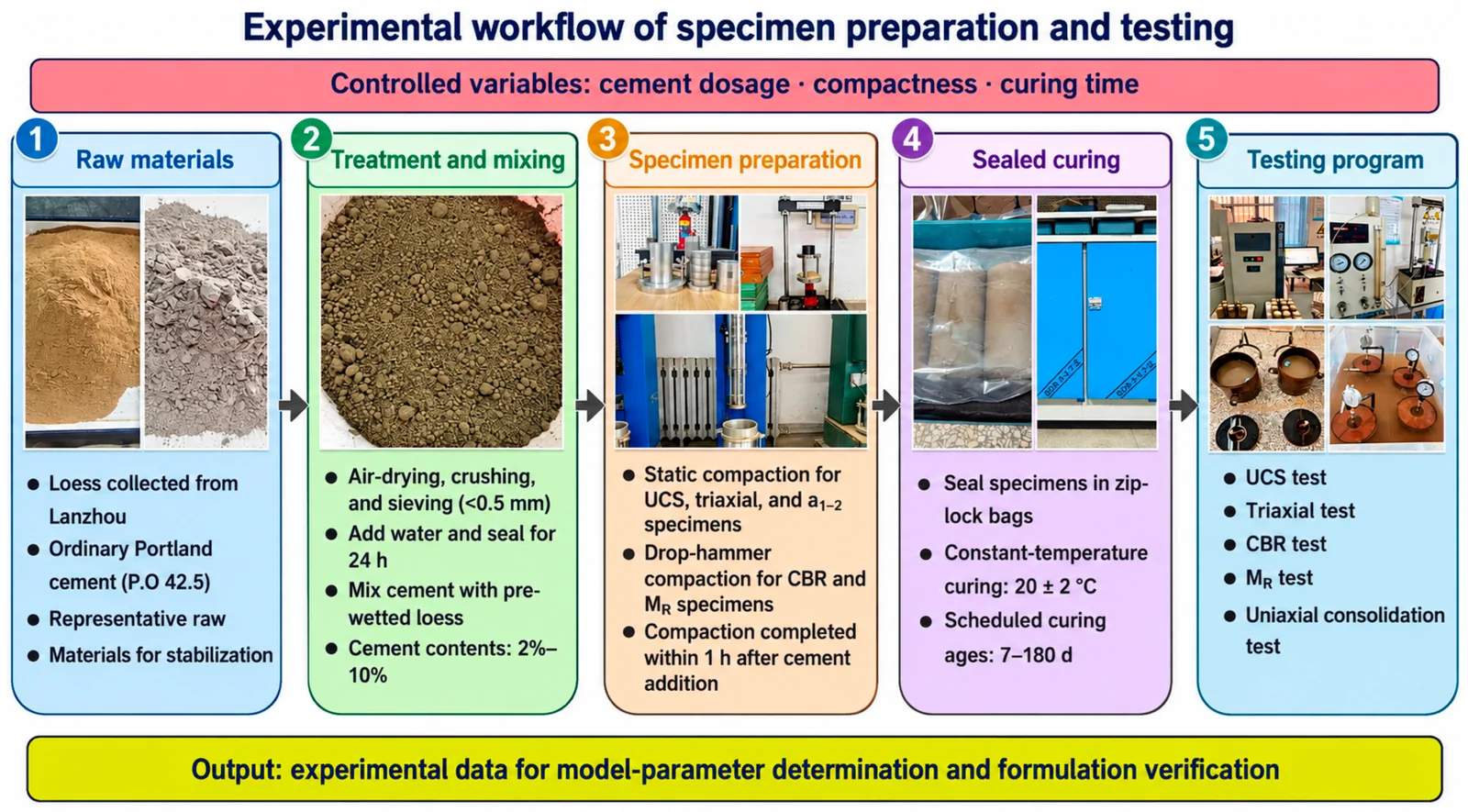
Experimental workflow of specimen preparation and testing.

The specimen dimensions and compaction methods used for different tests are summarized in Table 4. Specimens for UCS and triaxial tests were compacted statically in five layers, whereas specimens for CBR and M_R_ tests were prepared using a drop-hammer compaction method in three layers with 50 blows per layer. The compaction energy for CBR and M_R_ specimens was consistent with the standard heavy compaction procedure specified in JTG 3430-2020 [37]. Consolidation specimens were also prepared by static compaction. After preparation, all specimens were sealed in zip-lock bags and cured in a constant-temperature chamber at 20 ± 2 °C until the specified curing age. The overall workflow, including specimen preparation, constant-temperature curing, engineering performance testing, and data acquisition for model-parameter determination, is illustrated in Figure 2.

### 3.3. Testing Program

The experimental program was designed to provide the data required for determining the model parameters applicable to cement-stabilized loess, and for further evaluating the applicability and reliability of each formulation for different engineering indices. UCS tests were conducted to quantify the basic strength development of cement-stabilized loess and to provide a reference index for subsequent correlation analysis. Triaxial compression tests were used to determine the shear strength parameters, namely cohesion and internal friction angle, which are essential for slope stability evaluation. CBR and M_R_ tests were performed to characterize bearing capacity and deformation resistance for subgrade, road base, and foundation applications, while uniaxial consolidation tests were conducted to determine the compressibility coefficient *a*_1-2_, which is commonly used to assess the settlement tendency of subgrades and foundations.

All tests were conducted following JTG 3430-2020 [37]. The UCS, triaxial, and CBR tests were performed using strain-controlled loading equipment at a displacement rate of 1 mm/min. For the UCS test, loading was stopped when the peak load was reached and a post-peak decrease was observed, reflecting the brittle failure behavior of cement-stabilized specimens. The triaxial tests were terminated when the deviator stress became stable or the axial strain reached 20%. Confining pressures of 100, 200, 300, and 400 kPa were applied. For the M_R_ test, a lever-type pressure apparatus was used. The loading range specified for untreated soils was adjusted because the resilient deformation of stabilized specimens under low stress levels was relatively small and less stable. After repeated trial tests, six stress levels of 50, 100, 200, 300, 400, and 500 kPa were applied stepwise, with each load maintained for 1 min. For the uniaxial consolidation test, vertical pressures of 50, 100, 200, 400, and 800 kPa were adopted. For each mixture and curing condition in the triaxial tests, five specimens were prepared: four for testing under the confining pressures specified above and one as a spare. For all other tests, at least three replicate specimens were tested under each condition. The individual results deviated from their corresponding mean by no more than 10%, and the mean values were used for subsequent analysis. The coefficients of variation for UCS, CBR, MR, and uniaxial consolidation tests ranged from 0.63% to 2.71%, 0.69% to 3.09%, 0.38% to 5.52%, and 0.48% to 3.02%, respectively.

The test matrix for UCS, triaxial, and consolidation tests is summarized in Table 5. To examine the effects of cement dosage, specimens with cement contents of 2–10% were prepared at 0.96 *MDD* and tested at curing ages of 7 and 28 days. To evaluate the compactness effect, specimens with 4% cement, a representative dosage for loess improvement as discussed in Section 3.2, were prepared at different dry densities and tested at 7 and 28 days. To investigate curing-time development, specimens with 4% cement and 0.96 *MDD* were tested at 7, 14, 28, 60, 90, and 180 days. For the *CBR* and *M*_R_ tests, specimens were prepared using a fixed standard compaction procedure. Accordingly, the effects of cement dosage and curing period were emphasized for these two indices, whereas the independent effect of compactness on *CBR* and *M*_R_ was not examined in the present testing program.

## 4. Mechanical Performance Modeling and Assessment

### 4.1. Development and Assessment of UCS Models

*UCS* is one of the most widely used indices for evaluating the strength development and construction quality of stabilized soils. In pavement engineering, it is commonly adopted as a key quality-control parameter for road base and subbase materials [38]. In the present framework, *UCS* was used as the starting point for model calibration because it provides the most direct measure of cementation-induced strength development and offers a basis for examining parameter transferability to other engineering indices. Therefore, the *UCS* data were first used to examine the applicability of the modified formulations and to establish three-factor evaluation models incorporating cement dosage, compactness, and curing period.

At a selected reference curing age, the coupled effects of cement dosage and compactness on *UCS* were described using Equations (12)–(14). The 7-day and 28-day curing periods were selected because they are commonly used as representative ages for early strength assessment and engineering quality inspection. The corresponding *UCS* data were therefore used to determine the model parameters related to cement dosage and compactness before introducing the curing-time function.

**Figure 3:**
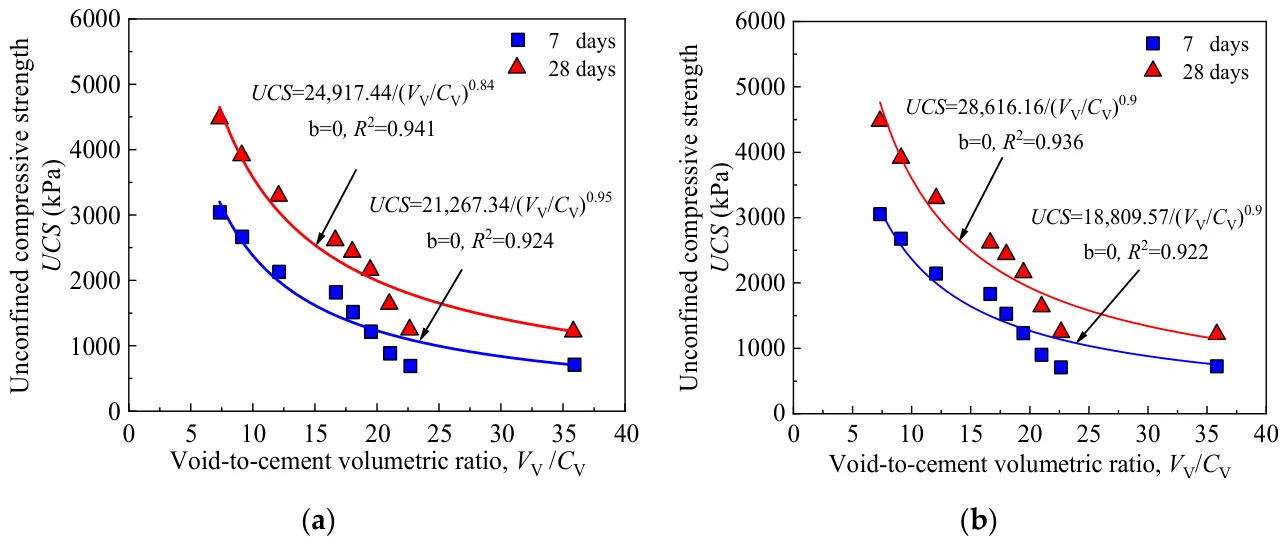
Relationships between *UCS* and V_V_/C_V_ for cement-stabilized loess: (**a**) fitting with age-specific x values; (**b**) fitting with the averaged x value.

For Equation (12), the effects of cement dosage and compactness were combined into the compound volumetric variable *V*_V_/*C*_V_. As shown in Figure 3a, *UCS* decreased with increasing *V*_V_/*C*_V_. This trend indicates that the mechanical performance of cement-stabilized loess is controlled by the competition between the positive filling and cementation effects of cement hydration and the adverse effect of porosity; when *V*_V_/*C*_V_ increases, the latter becomes dominant, leading to a weakened cemented soil skeleton. By setting parameter b to zero for model simplification, satisfactory fitting results were obtained when x was taken as 0.84 and 0.95 for the 7-day and 28-day data, respectively. The difference between these two values suggests that the sensitivity of *UCS* to the void–cement volumetric ratio varied with curing period. This age-dependent variation may be associated with the formation of cement hydration products, which can fill pores and strengthen interparticle bonding. However, *C*_V_ and *V*_V_ characterize the initial compacted state and do not directly quantify changes in hydration-product or pore volumes. The evolution of mechanical performance with curing time is described separately by Equation (20). It is worth noting that the agreement between the fitted curve and the experimental data becomes relatively poorer in the latter part of the *UCS-V*_V_*/C*_V_ relationship. This can be attributed to the non-proportional effects of cement dosage and initial porosity on *UCS*. To reduce the dependence of the model on a specific curing age and to obtain a generalized parameter value for subsequent model development, x was assigned the average of the fitted values obtained from the 7-day and 28-day data. As shown in Figure 3b, the adjusted model still provided acceptable agreement with the experimental results.

**Figure 4:**
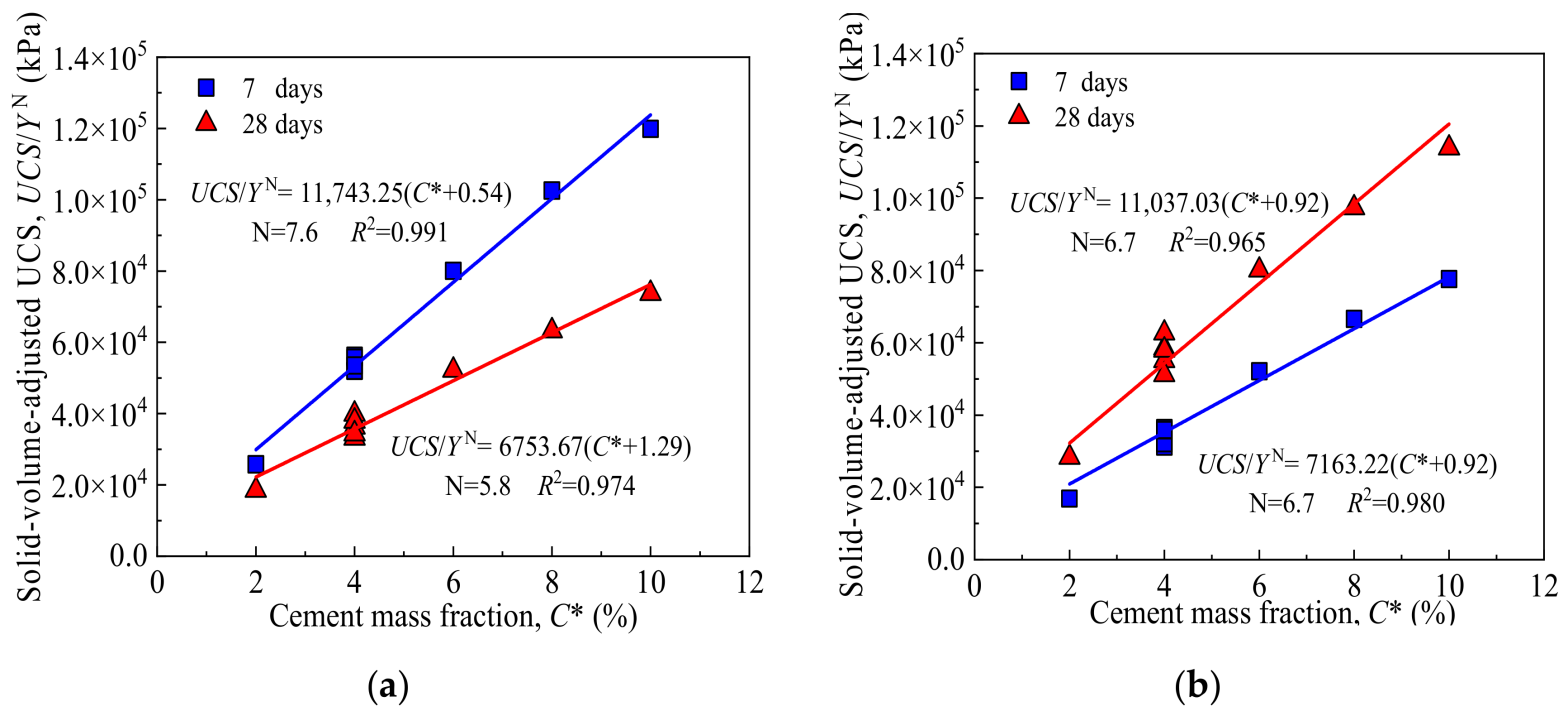
Relationships between *UCS*/*Y*^N^ and C* for cement-stabilized loess: (**a**) fitting with age-specific *C*_0_* and N values; (**b**) fitting with averaged *C*_0_* and N values.

For Equation (13), the cementation effect is represented by the cement content exceeding a threshold value, while the compactness effect is expressed through *Y*^N^. The parameter N reflects the sensitivity of *UCS* to the solid-volume contribution and void distribution. By transforming Equation (13) into a linear form, the parameters were determined from the relationships between *UCS*/*Y*^N^ and C*, as shown in Figure 4. The obtained N values were 7.6 and 5.8 for the 7-day and 28-day data, respectively, again indicating that the influence of compactness became less pronounced as curing proceeded. The threshold cement contents *C*_0_* were 1.29 and 0.54 for 7 and 28 days, respectively. This decrease implies that a relatively small cement dosage may require a longer curing period before its contribution to strength development becomes evident. For subsequent model development, the average values of *C*_0_* and N were adopted to obtain a generalized parameter set with reduced dependence on age-specific fitting results.

Equation (14) has a similar physical basis to Equation (12), as both formulations describe the balance between the positive contribution of cement volume and the adverse effect of initial void volume. However, their mathematical treatments are different. Equation (12) compresses *C*_V_ and *V*_V_ into a single compound ratio, whereas Equation (14) assigns independent power exponents to *C*_V_ and *V*_V_. This treatment allows the cementation effect and the void-structure effect to contribute to *UCS* development in a non-proportional manner, which is more consistent with the fitting behavior observed in the *UCS*–*V*_V_/C_V_ relationship.

**Figure 5:**
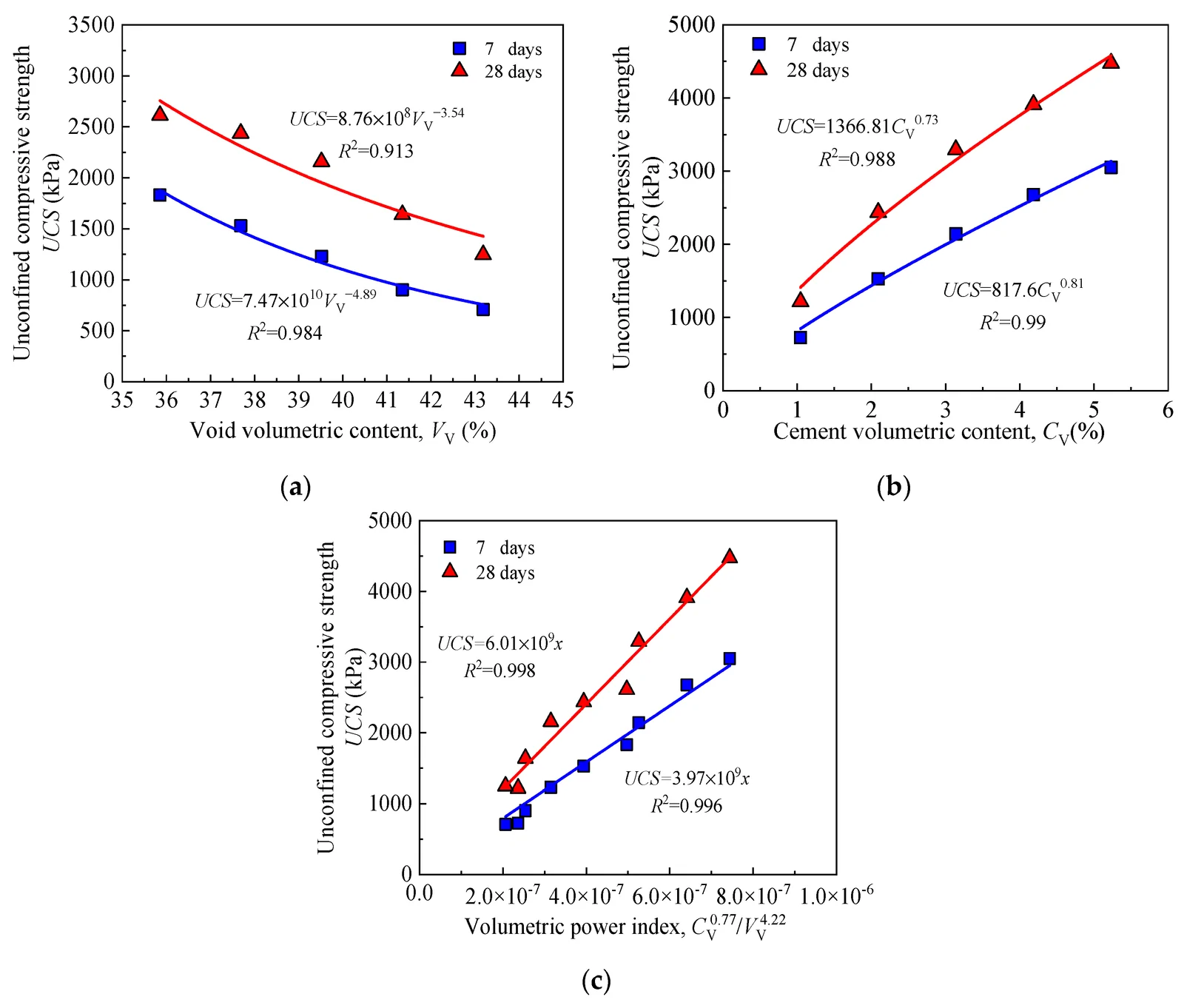
Application of Equation (14) to the *UCS* prediction of cement-stabilized loess: (**a**) *UCS*-*C*_V_ relationship; (**b**) *UCS*-*V*_V_ relationship; (**c**) *UCS*-*C*_V_^0^.^77^/*V*_V_^4.22^ relationship.

The parameters m and n in Equation (14) were determined based on the separate relationships between *UCS* and the two volumetric variables. As shown in Figure 5a and b, the effects of *C*_V_ and *V*_V_ on *UCS* can both be described by power functions for the 7-day and 28-day curing periods. Specifically, m reflects the contribution of cement volume to strength development through hydration-induced bonding and pore filling, whereas n characterizes the sensitivity of *UCS* to the initial void structure. The fitted results show that the absolute value of n at 7 days is greater than that at 28 days, indicating that *UCS* is more sensitive to the initial void structure during early curing. By contrast, the cement-related exponent m becomes more pronounced at 28 days, suggesting that the contribution of cement hydration gradually increases with curing period. This variation is consistent with the strength development mechanism of cement-stabilized loess. In the early curing stage, the soil skeleton formed by compaction provides the main load-transfer structure, and the negative influence of initial porosity is therefore significant. As curing proceeds, hydration products progressively fill pores, bridge particles, and strengthen interparticle bonding, thereby increasing the contribution of cement volume to *UCS* [39]. To reduce age-specific parameter dependence and to establish a generalized parameter set for the subsequent comprehensive *UCS* model, the average values of the 7-day and 28-day exponents were adopted, giving m = 0.77 and n = 4.22.

As shown in Figure 5c, the relationship between *UCS* and *C*_V_^0^.^77^/*V*_V_^4^.^22^ exhibits a clearer fitting trend than the *UCS*-*V*_V_/*C*_V_ relationship in Figure 3. This indicates that Equation (14) can better accommodate the different variation rates of cementation and porosity effects. Although Equation (14) involves one more exponent parameter than Equation (12), it substantially reduces the scatter caused by using a single ratio variable and thereby improves the reliability of *UCS* prediction. Therefore, Equation (14) offers a more generalized and physically interpretable model form for describing the coupled effects of cement dosage and compactness.

**Figure 6:**
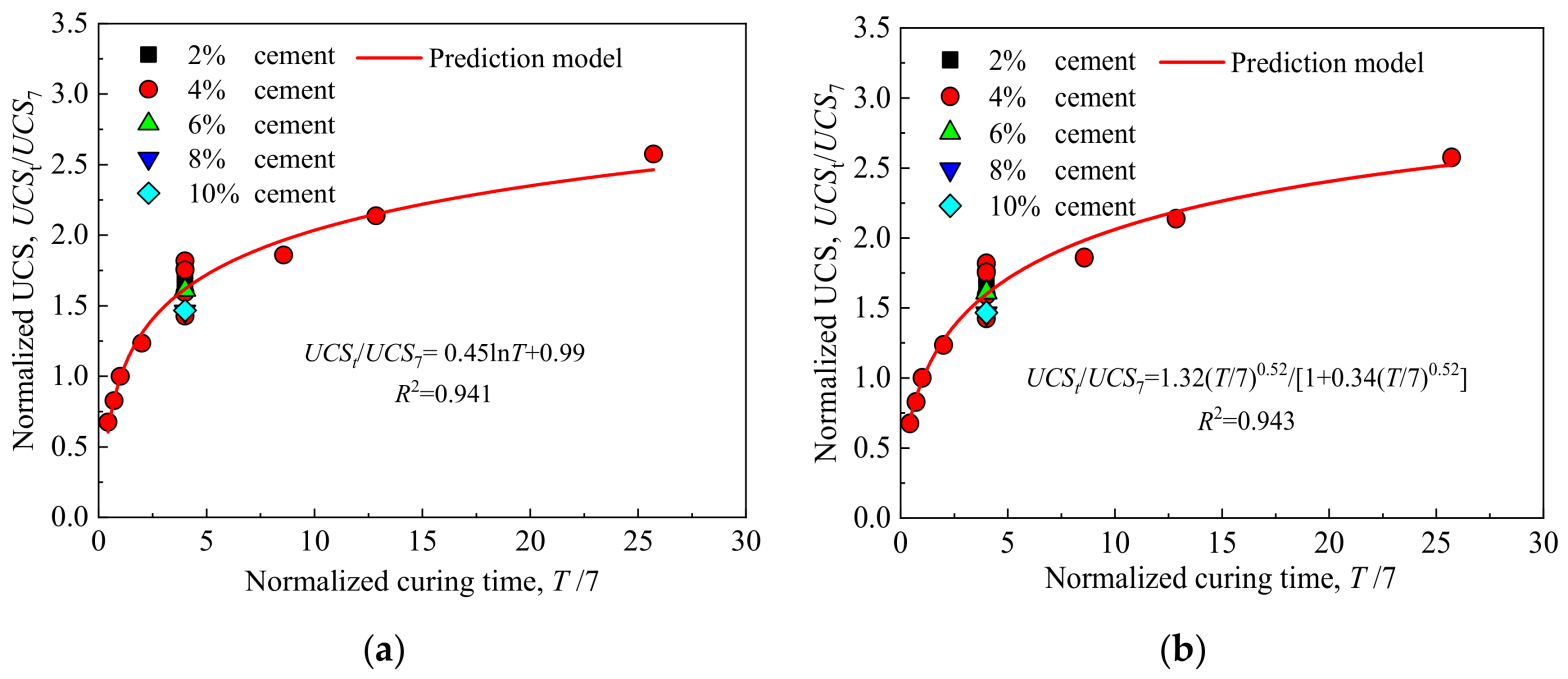
Relationships between normalized *UCS* and *T*: (**a**) logarithmic model, Equation (19); (**b**) hyperbolic model, Equation (20).

After the coupled effects of cement dosage and compactness were characterized at the reference curing ages, the curing-time effect on UCS was further examined using the normalized logarithmic and hyperbolic functions. As shown in Figure 6, the normalized *UCS* increased rapidly in the early curing stage and then gradually approached a slower growth rate with increasing curing period. Both the logarithmic and hyperbolic functions can describe the measured *UCS* development within the tested curing period. However, the two functions imply different long-term evolution patterns. The logarithmic function provides a convenient description of early-age strength growth, but it predicts a continuously increasing *UCS* with curing time. This implication is not fully consistent with the mechanical evolution of cement-stabilized loess, because the strength contribution from cement hydration should eventually approach a relatively stable state after sufficient curing. In contrast, the hyperbolic function contains an asymptotic form and can therefore describe both the rapid early-age increase and the gradual stabilization of *UCS* at later curing ages. Therefore, Equation (20) was considered more physically reasonable for representing the curing-time effect in long-term prediction.

Considering that the *UCS* of cement-stabilized loess develops rapidly during the early curing period and that 7-day *UCS* is commonly used as an important reference for opening road base and subbase layers to traffic [36], the 7-day UCS was selected as the reference value in this study. By coupling Equation (20) with the 7-day *UCS* models derived from Equations (12)–(14), the effects of cement dosage, compactness, and curing period were integrated into the comprehensive *UCS* evaluation models, as expressed in Equations (21)–(23).

(21) $$UCS\;(\mathrm{kPa})=\frac{18809.57}{{\left(V_{V}/C_{V}\right)}^{0.9}}\frac{1.32{\left(T/7\right)}^{0.52}}{1+0.34{\left(T/7\right)}^{0.52}}$$

(22) $$UCS\;(\mathrm{kPa})=\left[7163.22\left(C^{*}+0.92\right)Y^{6.7}\right]\frac{1.32{\left(T/7\right)}^{0.52}}{1+0.34{\left(T/7\right)}^{0.52}}$$

(23) $$UCS\;(\mathrm{kPa})=\left[3.97\times 10^{9}\frac{C_{V}^{0.77}}{V_{V}^{4.22}}\right]\frac{1.32{\left(T/7\right)}^{0.52}}{1+0.34{\left(T/7\right)}^{0.52}}$$

The agreement between the calculated and measured *UCS* values assessed using the average normalized difference (*AND*), as defined in Equation (24). In this equation, A_i_ and B_i_ represent the calculated and measured values of *UCS*, respectively, and n is the number of data points. The AND value provides a direct measure of the average relative deviation between the calculated and measured values and, when expressed as a percentage, is numerically equivalent to the mean absolute percentage error (MAPE). In addition, the mean absolute error (*MAE*) and root mean square error (*RMSE*) were introduced as complementary metrics to provide a more comprehensive assessment of model–data agreement.

**Figure 7:**
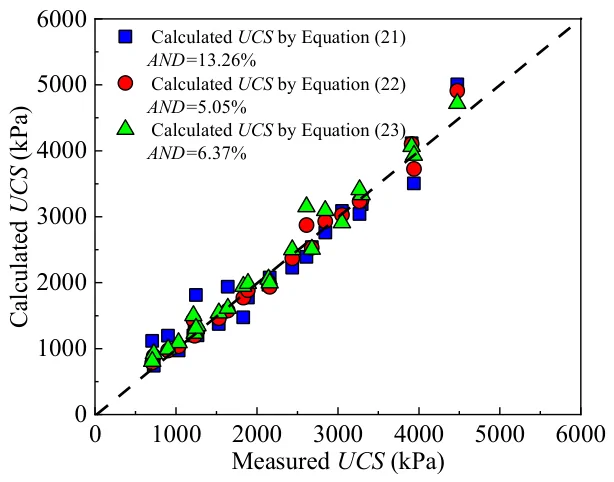
Comparison of calculated and measured *UCS* for cement-stabilized loess based on Equations (21)–(23).

As shown in Figure 7, the calculated *UCS* values obtained from Equations (21)–(23) are generally consistent with the measured results. The corresponding *AND* values are 13.26%, 5.05%, and 6.37%, respectively, while the corresponding *MAE*/*RMSE* values are 202.00/255.88, 110.52/150.38, and 130.24/173.24 kPa, respectively. These results indicate that all three comprehensive models can capture the combined effects of cement dosage, compactness, and curing period on *UCS* within an acceptable accuracy range. Among them, Equation (13) gives the lowest *AND* value, indicating better agreement with the experimental data when the threshold cement content and void-distribution sensitivity are explicitly considered. Equation (14) also shows a relatively low deviation from the measured data, indicating that the dual-power volumetric form can effectively describe the non-proportional effects of cement volume and initial void volume on *UCS*.

(24) $$AND=\frac{1}{n}\sum_{i=1}^{n}\left|\left(A_{i}-B_{i}\right)\right./\left.B_{i}\right|$$

### 4.2. Transferability of the Models to Other Mechanical Properties

The *UCS* analysis in Section 4.1 showed that the effects of cement dosage and initial void structure can be represented by several physically interpretable parameters (x, C_0_*, N, m and n). Since many engineering properties of stabilized soils are governed by similar cementation and skeleton-related mechanisms [27,28,40,41,42], it is reasonable to examine whether the parameter values obtained from *UCS* modeling can be transferred to other engineering indices. Such parameter transferability, if verified, would reduce the number of index-specific fitting parameters and improve the general applicability of the proposed evaluation framework.

Therefore, the proposed formulations were further extended to *c*, *φ*, *CBR*, *M*_R_, and *a*_1-2_. The following analysis focuses on whether the *UCS*-derived parameters can be directly transferred to these indices, or whether parameter recalibration or additional model terms are required because of index-specific response characteristics.

**Figure 8:**
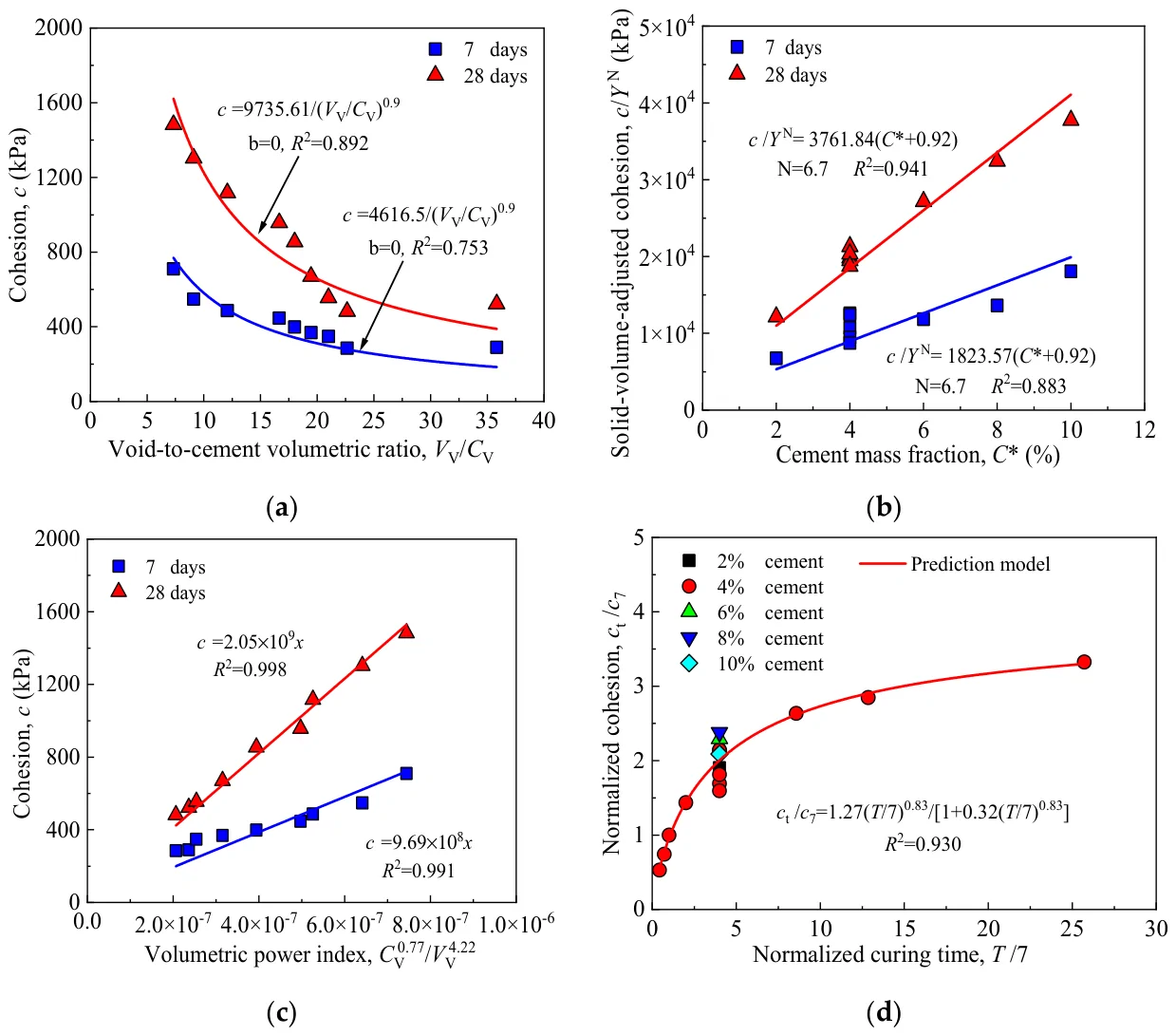
Evaluation of *c* in cement-stabilized loess using the proposed models: (**a**) Equation (12); (**b**) Equation (13); (**c**) Equation (14); (**d**) Equation (20).

The shear strength parameters were first examined to evaluate whether the *UCS*-derived parameters can be extended from a cementation-dominated strength index to indices involving both bonding and frictional resistance. For each test condition, the failure stresses obtained from the triaxial tests under confining pressures of 100, 200, 300, and 400 kPa were used to establish the corresponding Mohr–Coulomb failure envelope, from which *c* and the internal friction angle *φ* were determined. Because the compacted specimens were unsaturated and matric suction was not independently measured or controlled, *c* used herein should be interpreted as an apparent cohesion intercept under the prescribed testing conditions, incorporating the combined contributions of cementation and suction-related resistance, rather than effective cohesion *c*’ or undrained cohesion *c*_u_. As shown in Figure 8a–c, the *UCS*-derived parameters, including x = 0.9, C_0_* = 0.92, N = 6.7, m = 0.77, and n = 4.22, can still provide satisfactory fitting results for the cohesion data. This indicates that the effects of cement dosage and initial void structure on *c* can be described using the same parameter set as that used for *UCS*. In addition, the normalized hyperbolic function also captures the curing-time development of *c*, as shown in Figure 8d.

**Figure 9:**
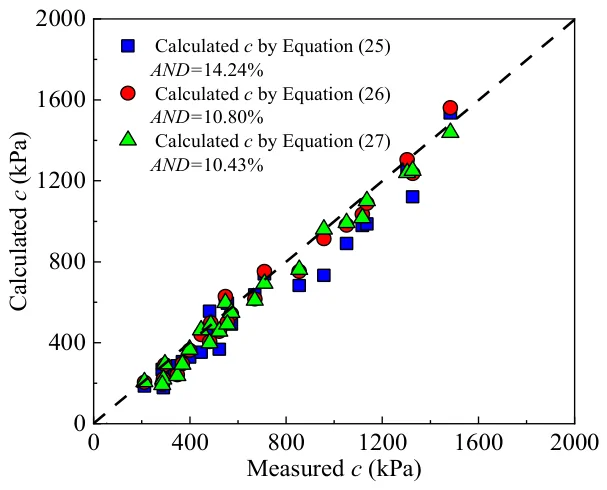
Comparison of calculated and measured *c* for cement-stabilized loess based on Equations (25)–(27).

By coupling the reference-age models with Equation (20), three-factor evaluation models for *c* were established, as expressed in Equations (25)–(27). The comparison between calculated and measured cohesion values is shown in Figure 9. The *AND* values of Equations (25)–(27) are 14.24%, 10.80%, and 10.43%, respectively, while the corresponding *MAE*/*RMSE* values are 87.57/106.49, 55.08/63.50, and 51.77/61.31 kPa, respectively, indicating that the proposed models can provide acceptable agreement with the measured *c* values.

(25) $$c\;(\mathrm{kPa})=\frac{4616.5}{{\left(V_{V}/C_{V}\right)}^{0.9}}\frac{1.27{\left(T/7\right)}^{0.83}}{1+0.32{\left(T/7\right)}^{0.83}}$$

(26) $$c\;(\mathrm{kPa})=\left[1823.57\left(C^{*}+0.92\right)Y^{6.7}\right]\frac{1.27{\left(T/7\right)}^{0.83}}{1+0.32{\left(T/7\right)}^{0.83}}$$

(27) $$c\;(\mathrm{kPa})=\left[9.69\times 10^{8}\frac{C_{V}^{0.77}}{V_{V}^{4.22}}\right]\frac{1.27{\left(T/7\right)}^{0.83}}{1+0.32{\left(T/7\right)}^{0.83}}$$

**Figure 10:**
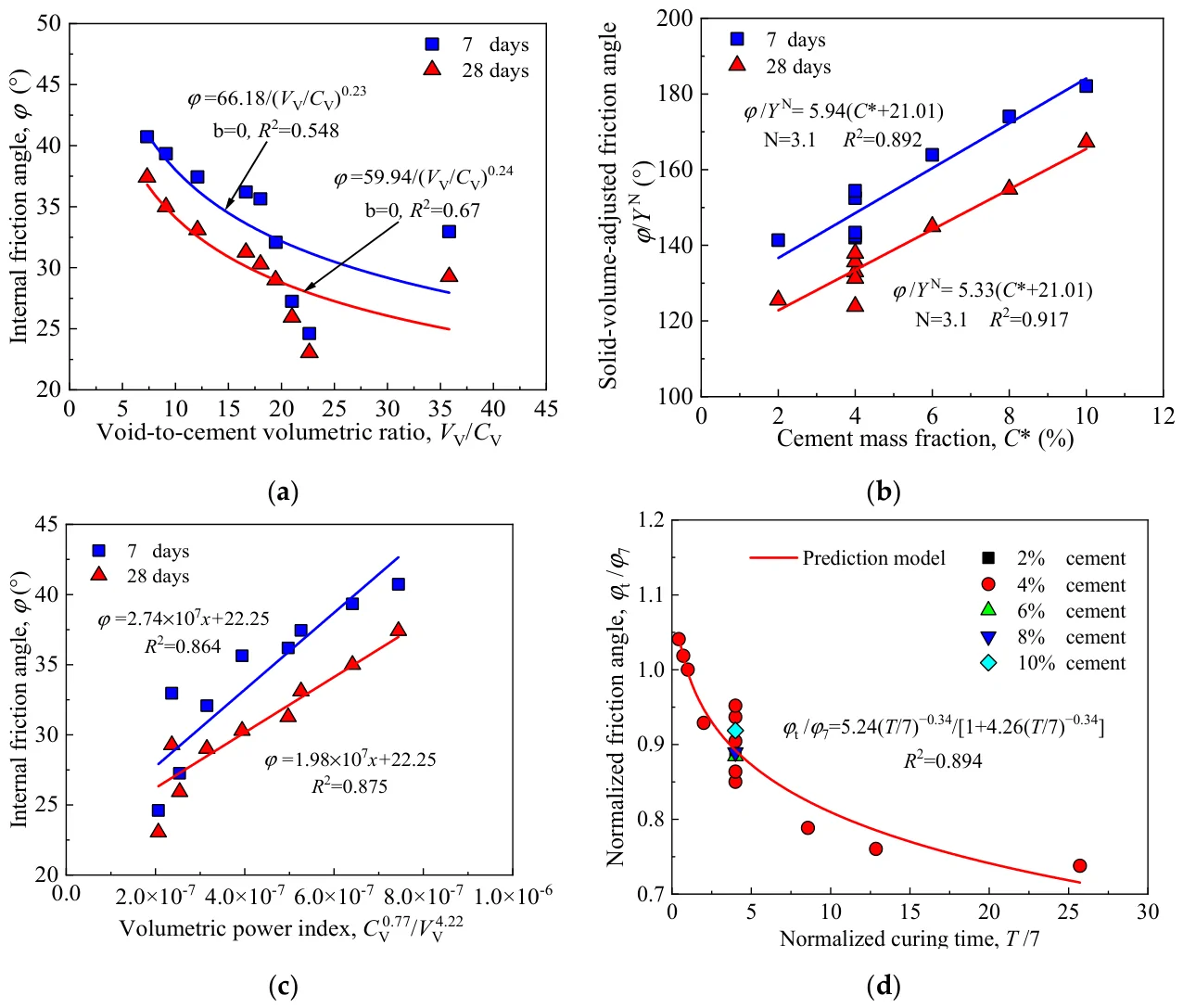
Evaluation of *φ* in cement-stabilized loess using the proposed models: (**a**) Equation (12); (**b**) Equation (13); (**c**) Equation (14); (**d**) Equation (20).

Unlike cohesion, the *φ* showed a weaker dependence on cementation-related parameters. As shown in Figure 10a, Equation (12) with the *UCS*-derived parameter x = 0.9 cannot satisfactorily describe the variation in *φ*. After regression analysis, the fitted x values for the 7-day and 28-day data were 0.23 and 0.24, respectively, but the corresponding correlation coefficients remained relatively low. This indicates that the compound variable *V*_V_/*C*_V_ is not suitable for describing *φ*, mainly because *φ* is governed more by particle arrangement, interparticle friction, and void distribution than by cementation strength alone. Therefore, when cement dosage and compactness are compressed into a single ratio variable, their different effects on *φ* cannot be adequately represented.

For Equation (13), the *UCS*-derived parameters N = 6.7 and C_0_* = 0.92 were also not directly applicable. After recalibration, N and C_0_* were determined as 3.1 and 21.01, respectively, as shown in Figure 10b. Compared with the *UCS* model, the smaller N value indicates that *φ* is less sensitive to compactness than *UCS*. Meanwhile, the much larger C_0_* value suggests that cement dosage has a weaker and less direct effect on *φ*. In the context of this empirical formulation, C_0_* = 21.01 implies that a relatively high cement-content threshold is required before cement addition can substantially alter the skeleton structure of cement-stabilized loess, such as particle contact, aggregation, and void arrangement, and thereby produce a noticeable change in *φ*. This is consistent with the experimental observation that the increase in *φ* was much smaller than the increase in *UCS* under the same variation in cement dosage or density.

For Equation (14), the *UCS*-derived exponents m = 0.77 and n = 4.22 remained applicable, but an intercept had to be introduced to represent the contribution of the untreated loess. As shown in Figure 10c, the added intercept was 22.25°, which is close to the internal friction angle of the untreated loess, 23.8°. This modification is physically reasonable because the improvement in *φ* caused by cement addition or porosity reduction is limited compared with its initial value. Therefore, unlike *UCS*, the baseline frictional resistance of the original loess cannot be ignored when evaluating *φ*.

The curing-time variation in *φ* is shown in Figure 10d. Differently from *UCS* and *c*, *φ* exhibited a slight decreasing trend with curing period. This behavior may be attributed to the fact that *φ* is mainly controlled by particle contact, gradation, and frictional interaction, which are less directly enhanced by cement hydration. During curing, cement hydration and particle aggregation may alter the contact structure and local particle arrangement, leading to a reduction in the measured friction angle. In addition, the triaxial-test-derived *φ* is sensitive to moisture variation; therefore, sealed curing was adopted in this study to maintain a relatively stable moisture condition and reduce curing-induced uncertainty. A more detailed discussion of the influence of curing condition on *φ* is provided in Section 4.

**Figure 11:**
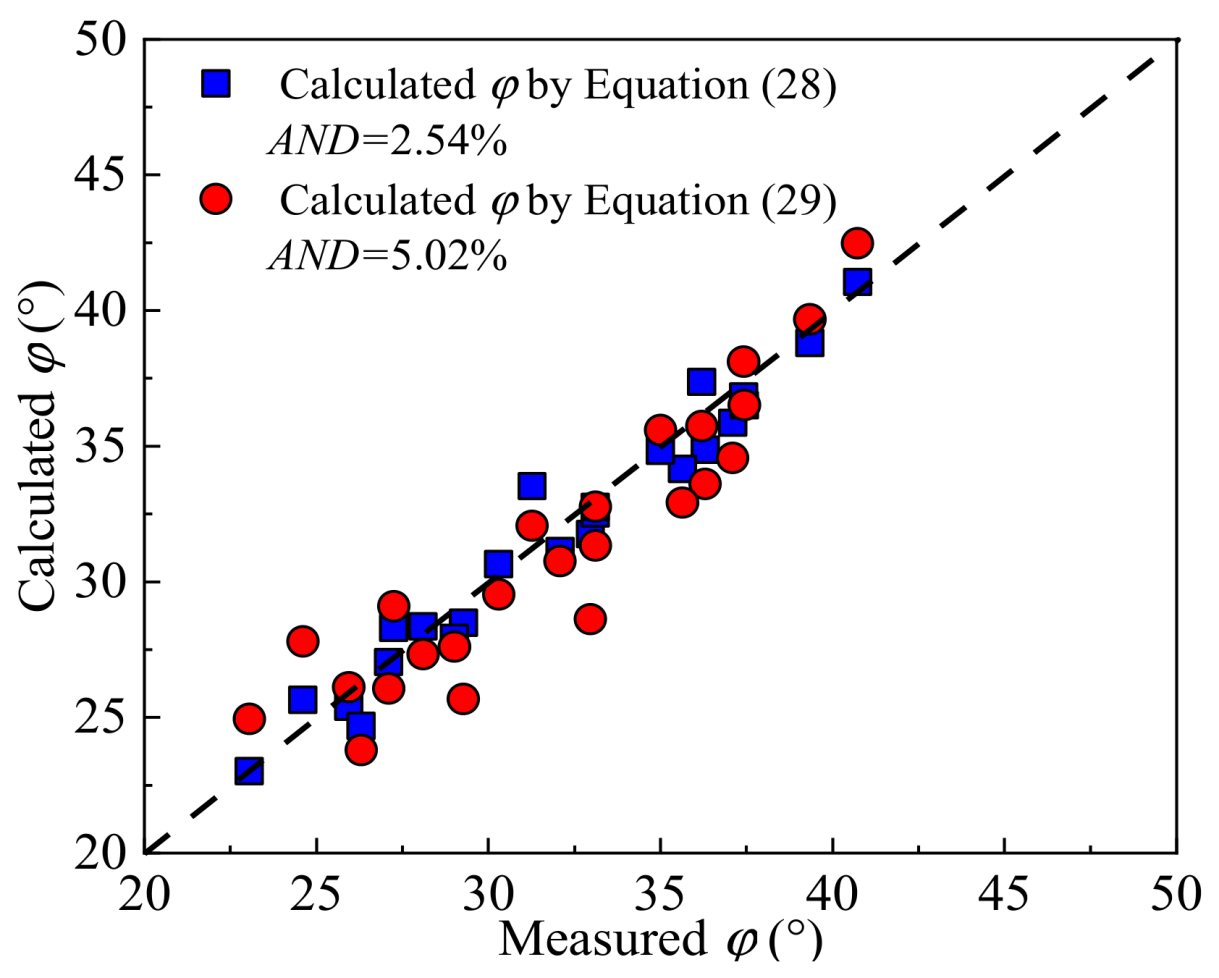
Comparison of calculated and measured *φ* for cement-stabilized loess based on Equations (28) and (29).

Based on the above analysis, Equation (12) was not used for *φ* prediction, whereas Equations (13) and (14) were adopted to establish the comprehensive evaluation models, as expressed in Equations (28) and (29). As shown in Figure 11, the calculated *φ* values agree well with the measured results, with *AND* values of 2.54% and 5.02%, respectively, and corresponding *MAE*/*RMSE* values of 0.83°/0.99° and 1.60°/1.95°, respectively. These results indicate that the threshold cement content model and the modified dual-power volumetric model can provide good agreement with the measured *φ* values, while the latter retains a clearer physical interpretation by incorporating the baseline frictional resistance of untreated loess.

(28) $$\varphi \;({}^{\circ})=\left[5.94\left(C^{*}+21.01\right)Y^{3.1}\right]\frac{5.24{\left(T/7\right)}^{-0.34}}{1+4.26{\left(T/7\right)}^{-0.34}}$$

(29) $$\varphi \;({}^{\circ})=\left[2.74\times 10^{7}\frac{C_{V}^{0.77}}{V_{V}^{4.22}}+22.25\right]\frac{5.24{\left(T/7\right)}^{-0.34}}{1+0.32{\left(T/7\right)}^{-0.34}}$$

**Figure 12:**
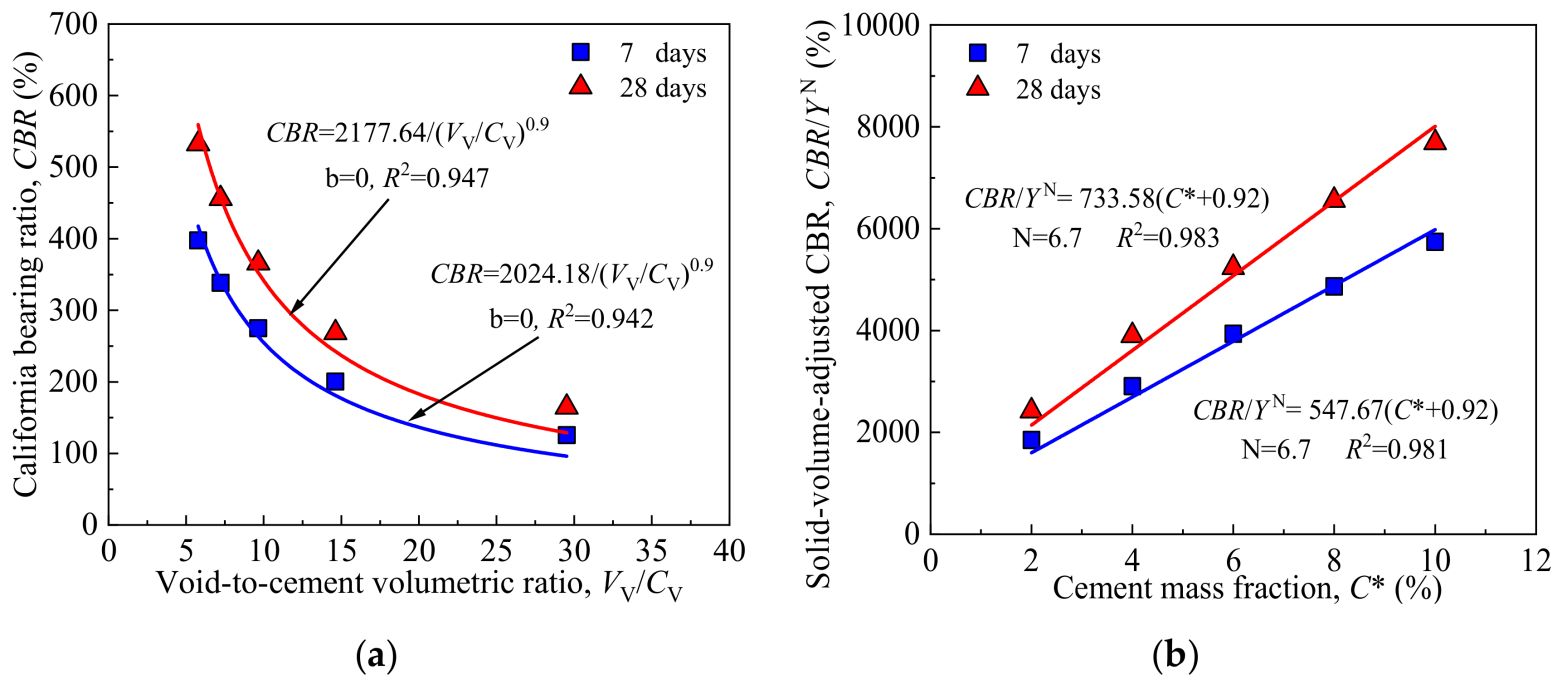

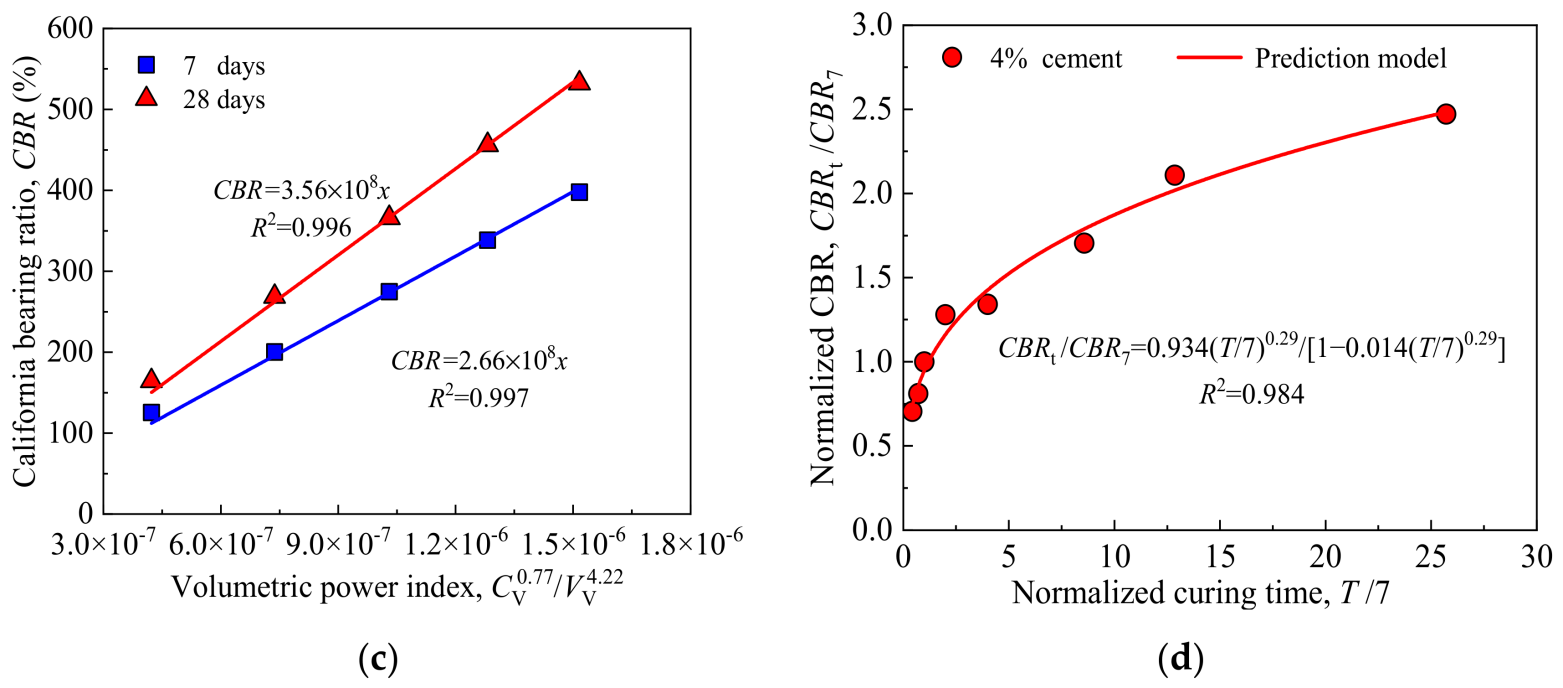
Evaluation of *CBR* in cement-stabilized loess using the proposed models: (**a**) Equation (12); (**b**) Equation (13); (**c**) Equation (14); (**d**) Equation (20).

**Figure 13:**
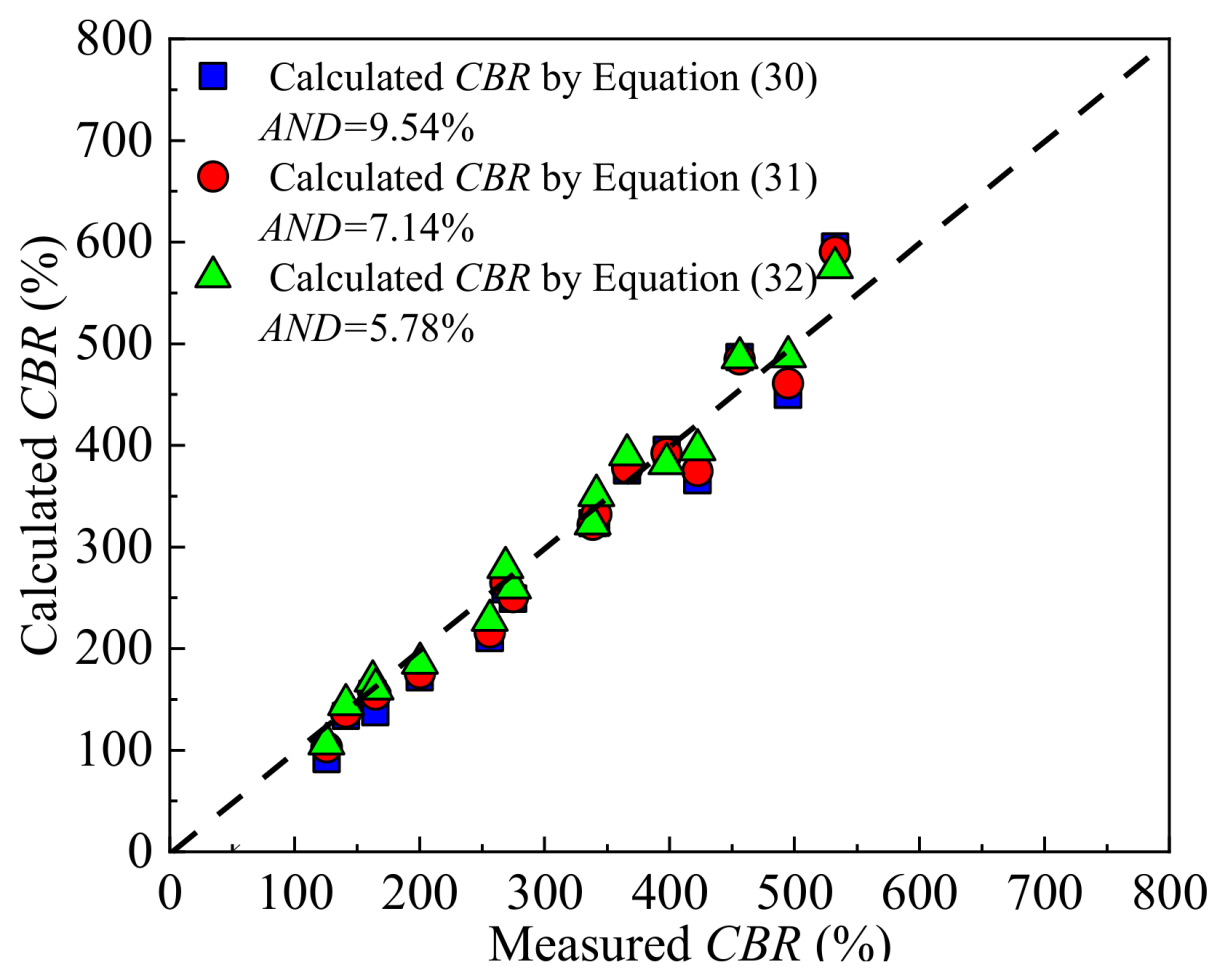
Comparison of calculated and measured *CBR* for cement-stabilized loess based on Equations (30)–(32).

*CBR* and *M*_R_ were further examined to evaluate the applicability of the proposed formulations to bearing capacity and deformation resistance. As shown in Figure 12a–c, the *UCS*-derived parameters, including x = 0.9, C_0_* = 0.92, N = 6.7, m = 0.77, and n = 4.22, remained applicable to *CBR* prediction. This indicates that the *CBR* of cement-stabilized loess responds to cement dosage and initial void structure in a manner similar to *UCS*. The curing-time development of *CBR* was also well described by the normalized hyperbolic function, as shown in Figure 12d. By coupling the reference-age models with Equation (20), the comprehensive *CBR* evaluation models were established, as expressed in Equations (30)–(32). The calculated *CBR* values agreed well with the measured results, with *AND* values of 9.54%, 7.14%, and 5.78% for Equations (30)–(32), respectively, as shown in Figure 13. The corresponding *MAE*/*RMSE* values are 26.63/32.15%, 21.45/26.91%, and 17.12/20.09%, respectively.

(30) $$CBR\;(\%)=\frac{2024.18}{{\left(V_{V}/C_{V}\right)}^{0.9}}\frac{0.934{\left(T/7\right)}^{0.29}}{1-0.014{\left(T/7\right)}^{0.29}}$$

(31) $$CBR\;(\%)=\left[547.67\left(C^{*}+0.92\right)Y^{6.7}\right]\frac{0.934{\left(T/7\right)}^{0.29}}{1-0.014{\left(T/7\right)}^{0.29}}$$

(32) $$CBR\;(\%)=\left[2.66\times 10^{8}\frac{C_{V}^{0.77}}{V_{V}^{4.22}}\right]\frac{0.934{\left(T/7\right)}^{0.29}}{1-0.014{\left(T/7\right)}^{0.29}}$$

**Figure 14:**
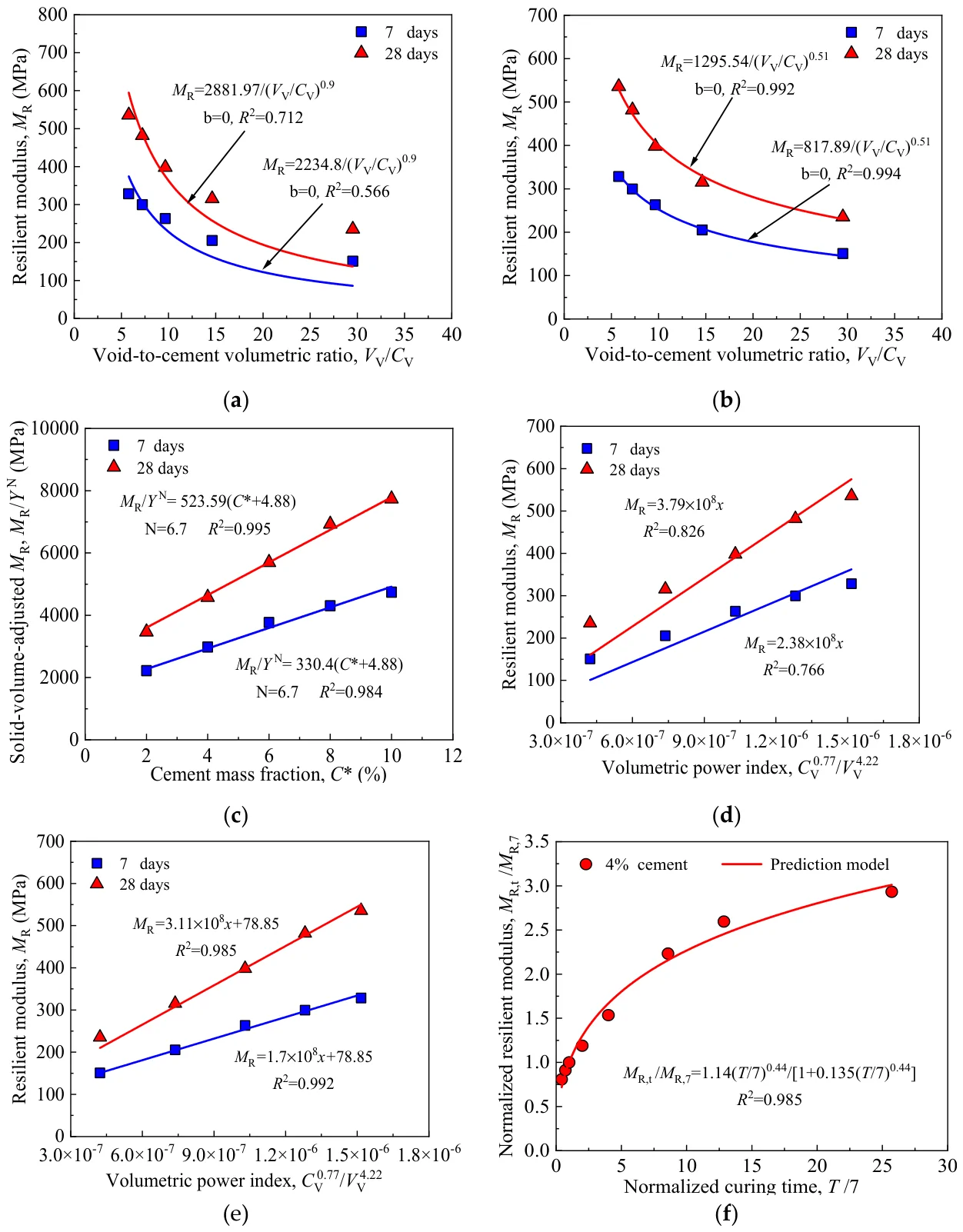
Evaluation of *M*_R_ in cement-stabilized loess using the proposed models: (**a**) Equation (12) with x = 0.9; (**b**) Equation (12) with x = 0.51; (**c**) Equation (13); (**d**) Equation (14); (**e**) Equation (14) with an intercept; (**f**) Equation (20).

Compared with *CBR*, *M*_R_ showed a weaker transferability of the *UCS*-derived parameters. As shown in Figure 14a, Equation (12) with the original parameter x = 0.9 did not provide satisfactory fitting results for *M*_R_. After recalibration, x was reduced to 0.51, as shown in Figure 14b, indicating that the *M*_R_ response was less sensitive to the cement-volume effect than *UCS*. As described above, this study did not specifically design different levels of densities for the specimens applied in CBR and M_R_ tests, and the *MDD* of the cement–loess mixtures changed only slightly with increasing cement dosage. Therefore, under a relatively consistent compactness condition, the decrease in x indicates that the influence of cement dosage on *M*_R_ is weaker than that on *UCS* for cement-stabilized loess. This is consistent with the experimental observation that the increase in *M*_R_ induced by cement addition was less pronounced than the increase in *UCS*.

For Equation (13), the *UCS*-derived values of N = 6.7 and C_0_* = 0.92 were also not directly applicable. When N was kept unchanged, C_0_* was recalibrated as 4.88, as shown in Figure 14c. The increase in C_0_* further indicates that a higher cement-content threshold is required before cement addition can produce an obvious improvement in resilient deformation resistance.

For Equation (14), the *UCS*-derived exponents m = 0.77 and n = 4.22 provided only moderate agreement with the *M*_R_ data when no intercept was included, as shown in Figure 14d. This is because the inherent resilient modulus of the untreated loess cannot be ignored when the improvement induced by cement stabilization is relatively limited. Therefore, an intercept equal to *M*_R,s_ = 78.85 MPa was introduced, corresponding to the measured *M*_R_ of the untreated loess. After this modification, Equation (14) showed improved agreement with the experimental data, as shown in Figure 14e. This treatment is consistent with the analysis of *φ*, where the baseline property of the untreated loess needs to be considered.

**Figure 15:**
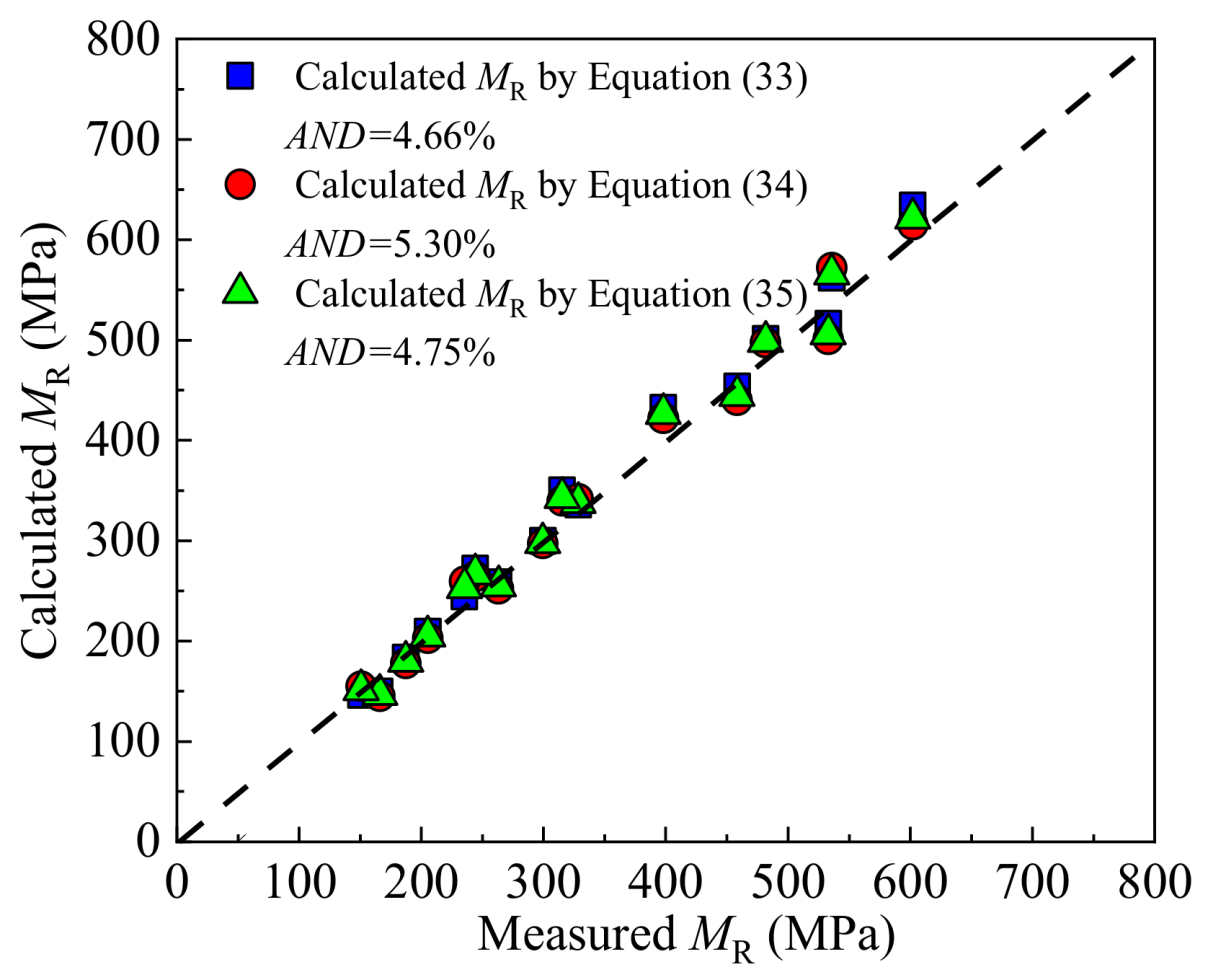
Comparison of calculated and measured *M*_R_ for cement-stabilized loess based on Equations (33)–(35).

The curing-time development of *M*_R_ was captured by Equation (20), as shown in Figure 14f. By incorporating the calibrated reference-age models and the normalized hyperbolic curing-time function, the comprehensive *M*_R_ evaluation models were obtained, as expressed in Equations (33)–(35). As shown in Figure 15, the calculated *M*_R_ values agreed well with the measured results, with *AND* values of 4.66%, 5.30%, and 4.75%, respectively, and corresponding *MAE*/*RMSE* values of 15.43/19.43, 16.95/19.53, and 15.69/18.55 MPa, respectively. These results indicate that the proposed formulations can be extended to the evaluation of *M*_R_. However, compared with *UCS*, *M*_R_ is less strongly governed by cementation-induced strength gain and is more influenced by the recoverable deformation characteristics of the original loess skeleton; therefore, parameter recalibration or baseline correction is required.

(33) $$M_{R}\;(\mathrm{MPa})=\frac{817.89}{{\left(V_{V}/C_{V}\right)}^{0.51}}\frac{1.14{\left(T/7\right)}^{0.44}}{1+0.135{\left(T/7\right)}^{0.44}}$$

(34) $$M_{R}\;(\mathrm{MPa})=\left[330.4\left(C^{*}+4.88\right)Y^{6.7}\right]\frac{1.14{\left(T/7\right)}^{0.44}}{1+0.135{\left(T/7\right)}^{0.44}}$$

(35) $$M_{R}\;(\mathrm{MPa})=\left[1.7\times 10^{8}\frac{C_{V}^{0.77}}{V_{V}^{4.22}}+78.85\right]\frac{1.14{\left(T/7\right)}^{0.44}}{1+0.135{\left(T/7\right)}^{0.44}}$$

*a*_1-2_ was finally examined to evaluate the applicability of the proposed formulations to settlement-related performance. Different from the strength- and stiffness-related indices discussed above, *a*_1-2_ evaluates compressibility, and its engineering implication is opposite: a lower *a*_1-2_ value indicates lower compressibility and a reduced settlement tendency. Therefore, the effects of cement dosage, compactness, and curing period on *a*_1-2_ are opposite to those on *UCS*, *c*, *CBR*, and *M*_R_.

**Figure 16:**
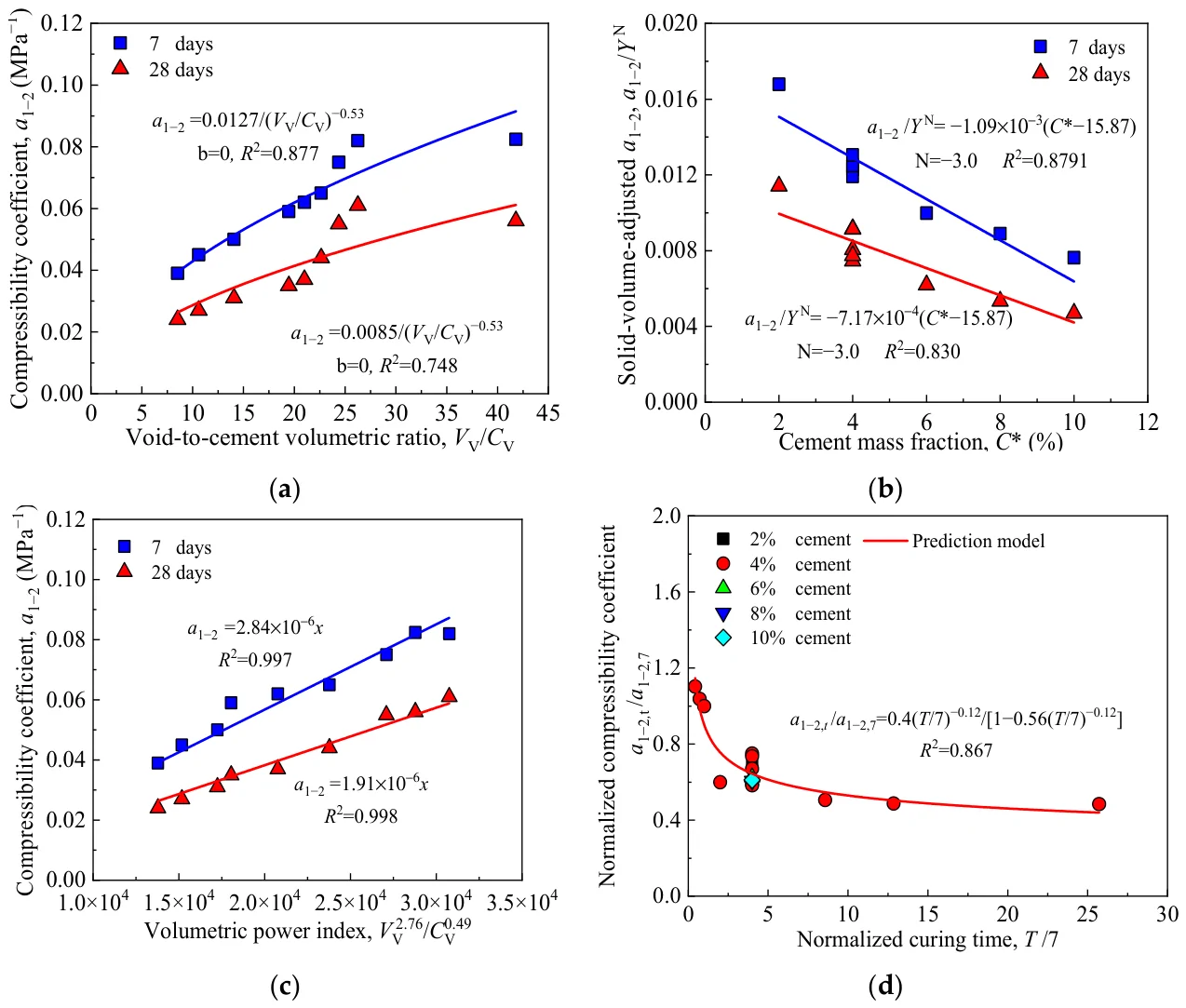
Evaluation of *a*_1-2_ in cement-stabilized loess using the proposed models: (**a**) Equation (12); (**b**) Equation (13); (**c**) Equation (14); (**d**) Equation (20).

As shown in Figure 16a–c, most parameters in the proposed formulations changed sign when they were applied to *a*_1-2_. This change is physically reasonable because increasing cement dosage, decreasing initial porosity, and extending curing period improve the stabilized soil structure and therefore reduce the compressibility coefficient. In terms of parameter magnitude, the increase in C_0_* and the decrease in m indicate that cement dosage has a weaker influence on *a*_1-2_ than on *UCS*. Similarly, the decreases in x, N, and n suggest that the sensitivity of *a*_1-2_ to initial void structure is also lower than that of *UCS* within the tested density range.

This behavior reflects the different mechanical nature of compressibility compared with strength-related indices. The coefficient *a*_1-2_ represents the gradual deformation of soil under sustained loading, whereas *UCS* and cohesion mainly describe the resistance to short-term failure. Cement hydration generates bonding and bridging between soil particles, which can markedly improve strength-related indices. However, the resistance to long-term compression deformation depends more strongly on the initial compacted soil skeleton, including particle contact density, void distribution, and load-transfer stability. In this study, the initial compaction degree of all specimens was higher than 85%, indicating that the soil skeleton had already formed a relatively dense load-bearing structure before cement hydration. Under this condition, the additional improvement provided by cementation was limited, and the reduction in *a*_1-2_ was mainly controlled by the initial compacted skeleton, with cement hydration acting as a supplementary reinforcing mechanism.

Among the three formulations, Equations (12) and (13) showed lower correlation coefficients for *a*_1-2_ than those obtained for the strength- and stiffness-related indices, whereas Equation (14) still provided satisfactory fitting results for both the 7-day and 28-day data. This indicates that the dual-power volumetric model is more suitable for describing the combined effects of cement volume and initial void volume on compressibility. The curing-time development of *a*_1-2_ was also well captured by Equation (20), as shown in Figure 16d.

**Figure 17:**
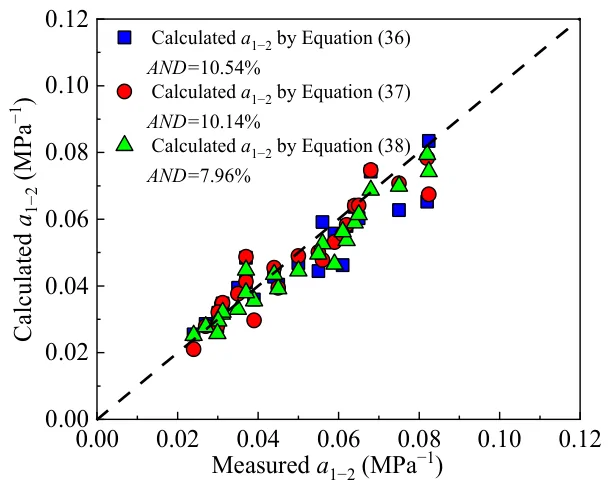
Comparison of calculated and measured a1-2 for cement-stabilized loess based on Equations (36)–(38).

By incorporating the reference-age models and the normalized hyperbolic curing-time function, comprehensive evaluation models for *a*_1-2_ were established, as expressed in Equations (36)–(38). As shown in Figure 17, the calculated *a*_1-2_ values agreed well with the measured results, with *AND* values of 10.54%, 10.14%, and 7.96%, respectively, and corresponding *MAE*/*RMSE* values of 0.0051/0.0068, 0.0046/0.0058, and 0.0039/0.0049 MPa^−1^, respectively. These results indicate that the proposed formulations can be used to evaluate the settlement tendency of subgrades and foundations filled with cement-stabilized loess. However, compared with strength-related indices, the prediction of *a*_1-2_ depends more strongly on the compacted soil skeleton, suggesting that improving compactness should be given priority when controlling settlement deformation in engineering practice.

(36) $$a_{1-2}{\;(\mathrm{MPa}}^{-1})=\frac{0.0127}{{\left(V_{V}/C_{V}\right)}^{-0.53}}\frac{0.4{\left(T/7\right)}^{-0.12}}{1-0.56{\left(T/7\right)}^{-0.12}}$$

(37) $$a_{1-2}{\;(\mathrm{MPa}}^{-1})=\left[-1.09\times 10^{-3}\left(C^{*}-15.87\right)Y^{-3}\right]\frac{0.4{\left(T/7\right)}^{-0.12}}{1-0.56{\left(T/7\right)}^{-0.12}}$$

(38) $$a_{1-2}{\;(\mathrm{MPa}}^{-1})=\left[2.84\times 10^{-6}\frac{V_{V}^{2.76}}{C_{V}^{0.49}}\right]\frac{0.4{\left(T/7\right)}^{-0.12}}{1-0.56{\left(T/7\right)}^{-0.12}}$$

Overall, the extension of the proposed formulations to different engineering indices shows that the *UCS*-derived parameters can be transferred effectively to *c* and *CBR*, indicating similar sensitivities of these indices to cement dosage and initial void structure. For *φ* and *M*_R_, however, partial parameter recalibration or baseline correction is necessary because the contribution of untreated loess cannot be ignored. For *a*_1-2_, the response mechanism differs more substantially from strength-related indices, and the initial compacted skeleton plays a more dominant role. Among the three formulations, Equation (14) shows better applicability to different engineering indices than Equations (12) and (13), because it separately represents the effects of cement volume and initial void volume and can maintain satisfactory prediction accuracy with limited parameter adjustment.

### 4.3. Evaluation Models Using 28-Day Reference Values

In addition to early-age evaluation, 28-day engineering properties are commonly used as reference values for quality inspection in practical engineering. Therefore, establishing evaluation models based on 28-day reference values can provide an additional basis for quality assessment and performance prediction of cement-stabilized loess.

In the preceding sections, the effects of cement dosage and compactness on the 28-day engineering indices were already described using the modified formulations. To extend these reference-age models to other curing periods, the normalized hyperbolic function in Equation (20) was further applied using the 28-day values as reference values. By coupling the 28-day reference-age models with Equation (20), comprehensive evaluation models based on 28-day reference values were obtained. The corresponding model parameters and *AND* values are summarized in Table 6. As shown in the table, the models based on 28-day reference values also provide satisfactory prediction accuracy for *UCS*, *c*, *φ*, *CBR*, *M*_R_, and a_1-2_. This result indicates that the proposed normalized framework is not limited to a specific reference curing age. Instead, it can be flexibly established using either 7-day or 28-day reference values according to the purpose of engineering evaluation.

From an engineering perspective, the 7-day reference models are more suitable for early-age strength assessment and construction-stage quality control, whereas the 28-day reference models are more consistent with conventional quality inspection practice. The availability of both reference-age systems improves the practical applicability of the proposed evaluation framework for design optimization, construction control, and post-construction performance prediction.

## 5. Discussion

The preceding analyses show that the proposed formulations can be extended from *UCS* to different engineering indices, but the degree of parameter transferability varies among indices. For *c* and *CBR*, the *UCS*-derived parameters can be directly retained, indicating that these indices have similar sensitivities to cement dosage and initial void structure. For *φ* and *M*_R_, partial recalibration or baseline correction is required because the contribution of untreated loess cannot be ignored. For *a*_1-2_, the response is fundamentally different from the strength- and stiffness-related indices because it reflects compressibility rather than resistance to strength failure or recoverable deformation. These differences indicate that the proposed framework should not be regarded as a simple set of empirical fitting equations; rather, it provides a structured way to identify how cementation development and skeleton densification contribute differently to each engineering property [43,44].

The parameter-transfer results also provide a basis for establishing simplified relationships between *UCS* and other engineering indices. Because *c*, *φ*, *CBR*, and *M*_R_ can be reasonably predicted using models that share part of the *UCS*-derived parameter structure, their values can be correlated with *UCS* at the same curing age. Based on the formulations with the best predictive performance, the relationships between *UCS* and these indices are summarized in Equations (39)–(42). Since the engineering properties of cement-stabilized loess tend to approach a relatively stable state with curing period, the relationships based on 28-day reference values are more representative for long-term engineering evaluation. In these equations, *φ*_s_ and *M*_R_,_s_ denote the *φ* and *M*_R_ of untreated loess, respectively, and the unit of *UCS* is kPa.

(39) $$c_{7}\;(\mathrm{kPa})=0.244UCS_{7}\;\mathrm{and}\;c_{28}\;(\mathrm{kPa})=0.341UCS_{28}$$

(40) $$\varphi_{7}\;(\circ )=6.9\times 10^{3}UCS_{7}+\varphi_{s}\;\mathrm{and}\;\varphi_{28}\;(\circ )=3.29\times 10^{3}UCS_{28}+\varphi_{s}$$

(41) $$CBR_{7}(\%)=0.067UCS_{7}\;\mathrm{and}\;CBR_{28}(\%)=0.059UCS_{28}$$

(42) $$M_{R,7}(\mathrm{MPa})=0.043UCS_{7}+M_{R,s}\;\mathrm{and}\;M_{R,28}(\mathrm{MPa})=0.052UCS_{28}+M_{R,s}$$

*a*_1-2_ requires separate discussion because its relationship with *UCS* is much weaker than that of the other indices. Settlement deformation is essentially associated with compression and rearrangement of the soil skeleton under sustained loading [24,26]. Cement hydration can attach to particle contacts, fill pores, and strengthen local bonding, but its contribution to reducing long-term compressibility is limited compared with its contribution to strength development. As discussed in Section 4.2, all specimens in this study had initial compaction degrees higher than 85%, indicating that a relatively dense load-bearing skeleton had already been formed before cement hydration. Under this condition, further reduction in *a*_1-2_ was mainly governed by the initial compacted skeleton, while cementation acted as a supplementary reinforcing mechanism. Therefore, in engineering practice, improving compactness should be prioritized when the settlement tendency of cement-stabilized loess is the main concern.

The variation in internal friction angle also deserves attention. In this study, *φ* showed a slight decreasing tendency with curing period, which differs from the monotonic increase or irregular variation reported in some previous studies [45,46,47,48]. This discrepancy is closely related to the fact that *φ* is more sensitive to particle arrangement, contact friction, and moisture condition than to cementation strength alone. During the preliminary stage of this study, three curing approaches were examined: direct exposure in the curing chamber, wrapping with plastic film, and sealing in zip-lock bags. Direct exposure led to unstable results because moisture exchange with the humid curing environment changed the water state of the specimens. Plastic-film wrapping reduced moisture disturbance but did not completely eliminate it. By contrast, sealing specimens in zip-lock bags effectively limited external moisture exchange and produced more consistent variations in *φ*. Therefore, the decreasing trend observed in this study should be interpreted under controlled moisture conditions, and the difference among reported *φ* trends may be partly attributed to differences in curing condition and test method.

**Figure 18:**
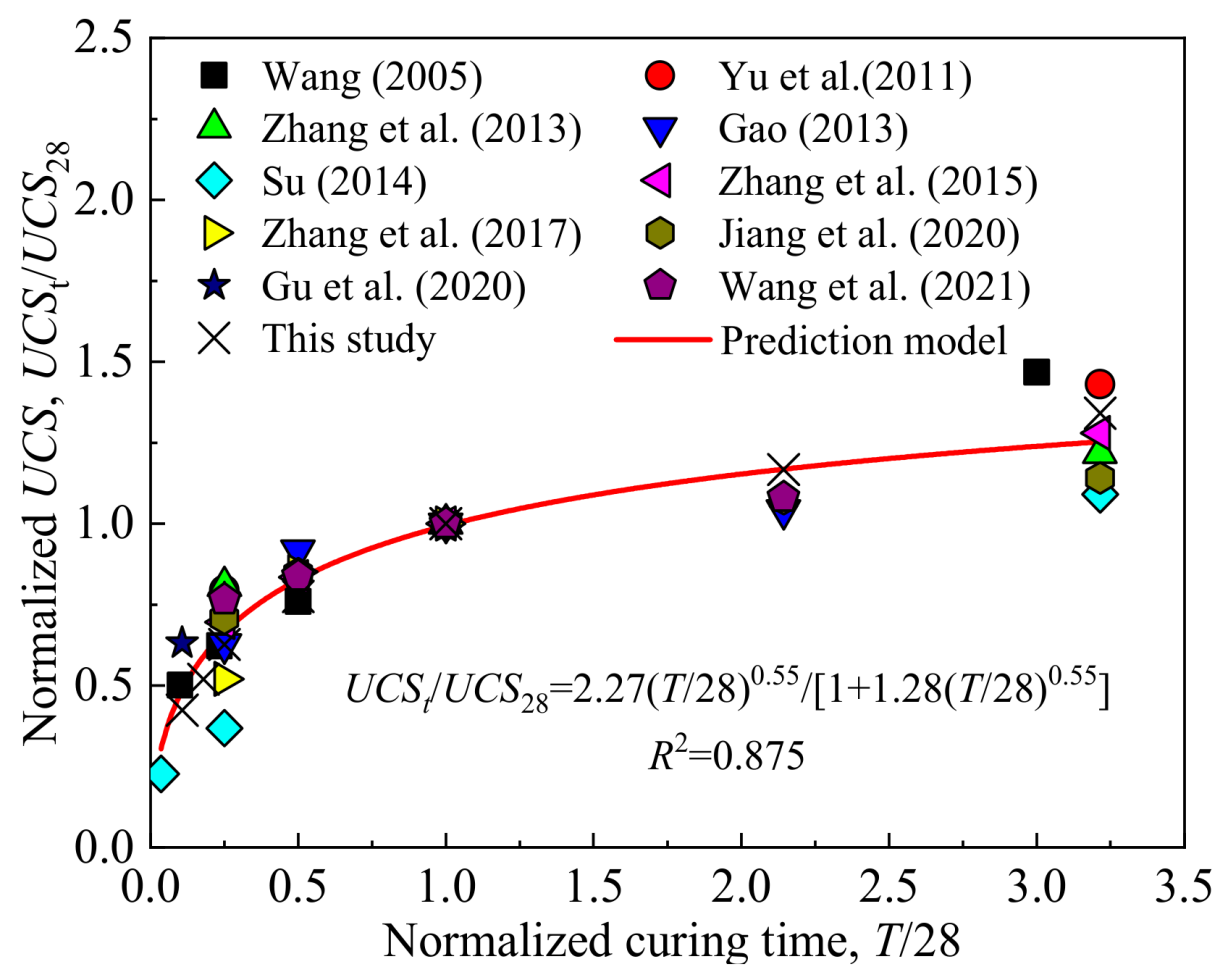
Time-dependent variation in normalized UCS for cement-stabilized loess based on literature datasets [11,29,49,50,51,52,53,54,55,56] and the present study.

To further improve the general applicability of the time-dependent model, additional *UCS* data for cement-stabilized loess were collected from previous studies, as summarized in Table 7. Considering engineering relevance and data consistency, the collected datasets were screened to include cement contents close to 4%. The normalization strategy was then applied to reduce the influence of differences in soil type, curing condition, and the absolute *UCS* values among different datasets. As shown in Figure 18, the normalized *UCS* development with curing period can be effectively described by Equation (20), leading to the generalized hyperbolic expression in Equation (43). This result indicates that the normalized hyperbolic function can capture the common time-dependent trend of cement-stabilized loess from different regions. Therefore, once the 28-day *UCS* is obtained, for example from laboratory specimens or in situ core samples, *UCS* at other curing ages can be estimated using Equation (43). Other engineering indices can then be further estimated through the *UCS*-based relationships in Equations (39)–(42).

(43) $$\frac{UCS_{t}}{UCS_{28}}=\frac{2.27{\left(T/28\right)}^{0.55}}{1+1.28{\left(T/28\right)}^{0.55}}$$

Overall, the proposed framework has two complementary engineering uses. The three-factor evaluation models based on Equations (12)–(14) and Equation (20) can be used during the design stage to select appropriate cement dosage and compactness for a target curing period. In contrast, the *UCS*-based relationships (Equations (39)–(42)) and the generalized hyperbolic model (Equation (43)) provide a practical route for construction-stage or post-construction quality prediction, because *UCS* can usually be obtained more conveniently than other engineering indices from laboratory specimens or in situ core samples. Among the three modified formulations, Equation (14) shows better applicability to different engineering indices because it separately represents the effects of cement volume and initial void volume and maintains satisfactory prediction accuracy with limited parameter adjustment. Nevertheless, the current models were calibrated using laboratory-prepared specimens under controlled curing conditions. Further validation using field-compacted materials, broader cement dosage ranges, and long-term environmental actions is still required before direct application to all engineering scenarios.

It should be noted that the laboratory dataset used for model calibration was also used to assess model performance. Therefore, the reported error statistics primarily reflect the agreement with the calibration data rather than independent predictive accuracy. Moreover, the literature-based analysis focuses on the normalized curing-time development of UCS and does not constitute independent validation of the complete multi-property framework. Further validation using independent laboratory and field datasets is required before broader engineering application.

The present framework uses the initial compacted void state and curing time to characterize mechanical development without explicitly tracking changes in moisture content or density during curing. Cement hydration contributes to strength development by filling pores and reinforcing interparticle contacts. Meanwhile, moisture content not only affects cement hydration but may also influence interparticle bonding and suction-related resistance. As water content and dry density at testing were not measured separately in this study, future work will monitor these parameters at different curing ages to further clarify the influence of moisture state on mechanical performance and improve the applicability of the proposed models under varying moisture conditions.

## 6. Conclusions

This study developed a porosity-based modeling framework to relate cement content, compacted void state, and curing time to the mechanical performance of compacted cement-stabilized loess. The main conclusions are as follows:

(1) The mechanical response of cement-stabilized loess can be effectively represented using cement volumetric content and initial void volumetric content as material-state variables. Among the three formulations, Equation (13) provided the lowest prediction error for *UCS*, whereas the dual-power volumetric model, Equation (14), showed better overall transferability across different mechanical properties because it separately accounts for the contributions of cement volume and void volume.

(2) The normalized hyperbolic function provided a physically reasonable description of curing-time evolution. Compared with the logarithmic function, its asymptotic form better reflects the tendency of cement-stabilized loess to approach a relatively stable mechanical state with prolonged curing. Coupling this function with the reference-age models enabled cement dosage, compacted void state, and curing period to be incorporated within a unified modeling framework.

(3) The transferability of *UCS*-derived parameters depended on the mechanical property considered. The *UCS*-derived parameters were directly applicable to *c*. For *CBR*, satisfactory prediction was obtained under fixed standard compaction, although the independent compactness effect remains to be verified. *φ* and *M*_R_ required parameter recalibration or baseline correction, whereas *a*_1-2_ showed a stronger dependence on the initial compacted soil skeleton within the investigated density range.

(4) Both 7-day and 28-day reference values could be incorporated into the normalized framework, demonstrating that the model formulation is not restricted to a single reference curing age. Simplified relationships between *UCS* and *c*, *φ*, *CBR*, and *M*_R_ further provide a convenient means of comparing the curing-dependent evolution of different mechanical properties.

(5) A generalized hyperbolic expression was further developed using literature datasets of cement-stabilized loess from different regions. With 28-day *UC*S as the reference value, this model enables *UCS* at other curing periods to be estimated through a unified time-dependent expression, extending the applicability of the time-dependent relationship across the literature datasets considered. Combined with the simplified *UCS*-based relationships for *c*, *φ*, *CBR*, and *M*_R_, the generalized model provides a practical basis for multi-property estimation. Further validation using field-compacted materials and long-term environmental conditions is required before application to post-construction performance assessment.

## Figures and Tables

**Table 1:** Summary of strength evaluation models.

No.	Strength Evaluation Models	Type of Stabilized Soil	References
(1)	$q_{u}\left(C_{c},W,t_{0}\right)=k_{1}k_{3}q_{u}(C_{c0},W_{0},t_{0})W^{-k_{2}}C_{c}^{k_{4}}$	Cement-stabilized high-water-content clay	[15]
(2)	$q_{t}=q_{t0}+K\log\left(t/t_{0}\right)$	Cement-stabilized soil	[16]
(3)	$q_{u}=\frac{K\left(c-c_{0}\right)}{{\left(G_{s}w/100+1\right)}^{2}}$	Cement-stabilized dredged soil	[17]
(4)	$\left\{\frac{q_{u{\left(w_{c}/c\right)}_{1,t}}}{q_{u{\left(w_{c}/c\right)}_{0,28}}}\right\}={\left[\frac{{\left(w_{c}/c\right)}_{0,28}}{{\left(w_{c}/c\right)}_{1,t}}\right]}^{B}\left(E+F\ln t\right)$	Cement-stabilized high-water-content clay	[18]
(5)	$q_{u}=\frac{a}{{\left(\frac{w_{c}}{c}\right)}^{x}}+b$	Cement-based solidification soil	[19]
(6)	$q_{u}=K_{c}\left(c^{*}-c_{0}^{*}\right)Y^{N}$	Cement-stabilized marine clays	[20]
(7)	$q_{u}=\exp\left(c_{1}\right){\left(\frac{w^{\prime }}{w_{L}}\right)}^{-c_{2}}\times t^{b_{1}\ln\left(c^{*}-b_{2}\right)+b_{3}}$	Cement-stabilized dredged soil	[21]
(8)	$q_{u}=a{\left[\frac{\eta }{{\left(B_{\mathrm{iv}}\right)}^{x}}\right]}^{-\alpha }$	Dispersive clay stabilized with distinct binders	[22]
(9)	$q_{u}=\frac{x}{y^{\left(w_{c}/C\right)}}\ln\left(t\right)=\frac{a+b\left(s/C\right)}{y^{\left(w_{c}/C\right)}}\ln\left(t\right)$	Cement-stabilized clay	[23]
(10)	$q_{u}=\frac{q_{u，0}}{{\left[{\left(W/C\right)/\left(W/C\right)}_{0}\right]}^{\alpha }}\frac{\beta {\left(t/7\right)}^{\gamma }}{1+\eta {\left(t/7\right)}^{\gamma }}$	Composite-binder-stabilized marine soft clay	[24]
(11)	$q_{u}=\frac{A_{w}^{m}}{C_{w}^{n}}\left[a\ln t+b\right]$	Cement-stabilized marine clay	[25]

Note: For all the equations listed in Table 1, *q*_u_ denotes *UCS*, *t* refers to the curing period, *w*_c_/*c* in Equations (4) and (5), *w*_c_/*C* in Equation (9) and *W*/*C* in Equation (10) are water–cement ratios, and the following symbols, k_1_, k_2_, k_3_ and k_4_ in Equation (1), K in Equations (2) and (3), B, E and F in Equation (4), a and b in Equations (5), (8), (9) and (11), x in Equations (5), (8) and (9), N in Equation (6), b_1_, b_2_ and b_3_ in Equation (7), α, β and γ in Equation (10), and m and n in Equation (11) are fitting parameters. The explanations for other physical parameters are as follows. For Equation (1): *C*_c_ and *W* mean the cement dosage and moisture content, respectively, and *C*_c0_, *W*_0_ and *t*_0_ refer to the specified cement dosage, moisture content and curing time, respectively. For Equation (2): *q*_t_ and q_to_ refer to *UCS* cured for *t* and *t*_0_ days, respectively. For Equation (3): *c* refers to the mass ratio of cement to soil; *c*_0_ is the minimum cement content required to trigger strength development; and *w* is water content. For Equation (4): The subscripts 0 and 1 mean the reference value and calculated value, respectively. For Equation (6): *K*_c_ refers to the strength increment coefficient; c_*_ means the mass ratio between cement and soil; *c*_0_* denotes the minimum cement content required to trigger strength development; and *Y* is the volumetric content of solid particles. For Equation (7): c_1_ and c_2_ refer to strength parameters normalized to water content related to 1 h curing after mixing with cement; *w*’/*w*_L_ is the normalized initial water content. For Equation (8): *B*_iv_ refers to the volumetric content of binder materials. For Equation (9): *s*/*C* denotes the ratio of soil mass to cement mass. For Equation (11): *A*_w_ and *C*_w_ refer to the cement content and total water content, respectively.

**Table 2:** Physical and engineering properties of loess.

Property	Value
Particle size	≤0.5 mm
Liquid limit, *LL*	27.2%
Plastic limit, *PL*	14.5%
Plasticity index *PI*	12.7%
Specific gravity, *Gs*	2.71
Soil classification	CL
Cohesion (kPa)	15.8
Internal friction angle (°)	23.8

**Table 3:** *MDD* and *OWC* for each mixture.

Cement Content (%)	*MDD* (g/cm^3^)	*OWC* (%)
2	1.78	14.8
4	1.78	15.2
6	1.79	15.6
8	1.79	16.1
10	1.79	16.4

**Table 4:** Specimen dimensions and preparation methods.

Test	Size (Diameter × Height)	Preparation Method
UCS test and triaxial test	61.8 mm × 125 mm	Static compaction in five layers
CBR test and M_R_ test	152 mm × 120 mm	Dynamic compaction in three layers, with 50 blows per layer
Uniaxial consolidation test	79.8 mm × 20 mm	Static compaction

**Table 5:** Experimental matrix for UCS, triaxial, and uniaxial consolidation tests.

Case	Purpose	Cement Content (%)	Density (g/cm^3^)	Curing Period (Day)
1	Cement content effect	2	0.96 *MDD*	7, 28
2		4		
3		6		
4		8		
5		10		
6	Dry density effect	4	0.96 *MDD* + 0.05	7, 28
7			0.96 *MDD* − 0.05	
8			0.96 *MDD* − 0.1	
9			0.96 *MDD* − 0.15	
10	Curing period effect	4	0.96 *MDD*	14
11				60
12				90
13				180

**Table 6:** Parameters and error metrics of the comprehensive evaluation models using 28-day reference values.

Engineering Indices	Models	Parameters	*AND* (%)	*MAE*	*RMSE*
*UCS*	Equation (12)	a = 28,616.16, x = 0.9, b = 0	13.82	244.09	279.67
	Equation (13)	K_1_ = 11,037.03, C_0_* = 0.92, N = 6.7	7.20	133.44	157.67
	Equation (14)	K_2_ = 6.01 × 10^9^, m = 0.77, n = 4.22	6.99	131.55	163.88
	Equation (20)	*α* = 1.32, *β* = 0.36, *γ* = 0.42	/	/	/
*c*	Equation (12)	a = 9735.61, x = 0.9, b = 0	13.10	82.42	103.50
	Equation (13)	K_1_ = 3761.84, C_0_* = 0.92, N = 6.7	10.89	60.24	69.36
	Equation (14)	K_2_ = 2.05 × 10^9^, m = 0.77, n = 4.22	9.36	48.08	54.96
	Equation (20)	*α* = 2.07, *β* = 1.13, *γ* = 0.81	/	/	/
*φ*	Equation (13)	K_1_ = 5.33, C_0_* = 21.01, N = 3.1	2.78	0.93	1.28
	Equation (14)	K_2_ = 1.98 × 10^7^, m = 0.77, n = 4.22	4.82	1.33	1.93
	Equation (20)	*α* = 0.1, *β* = −0.91, *γ* = −0.01	/	/	/
*CBR*	Equation (12)	a = 2177.64, x = 0.9, b = 0	3.94	26.27	32.23
	Equation (13)	K_1_ = 733.58, C_0_* = 0.92, N = 6.7	1.75	21.10	27.01
	Equation (14)	K_2_ = 3.56 × 10^8^, m = 0.77, n = 4.22	1.60	17.37	20.16
	Equation (20)	*α* = 1.048, *β* = −0.015, *γ* = 0.29	/	/	/
*M*_R_	Equation (12)	a = 1295.54, x = 0.51, b = 0	12.45	20.40	26.77
	Equation (13)	K_1_ = 523.59, C_0_* = 4.88, N = 6.7	5.43	20.61	24.93
	Equation (14)	K_2_ = 3.11 × 10^8^, m = 0.77, n = 4.22	7.19	21.70	26.91
	Equation (20)	*α* = 1.36, *β* = 0.248, *γ* = 0.44	/	/	/
*a*_1-2_	Equation (12)	a = 0.0085, x = −0.53, b = 0	14.00	0.0065	0.0078
	Equation (13)	K_1_ = −7.17 × 10^−4^, C_0_* = −15.87, N = −3.0	11.33	0.0052	0.0066
	Equation (14)	K_2_ = 1.91 × 10^−6^, m = 0.49, n = 2.76	9.04	0.0039	0.0050
	Equation (20)	*α* = 0.4, *β* = −0.56, *γ* = −0.12	/	/	/

Note: The units of *MAE* and *RMSE* are the same as those of the corresponding engineering indices.

**Table 7:** Literature datasets of cement-stabilized loess used for generalized model development.

Data Sources	Liquid Limit (%)	Plastic Limit (%)	Cement Content (%)	Region
Wang [49]	26.7	11.2	3.8	Xi’an City, Shannxi Province
Yu et al. [50]	39.00	23.8	5	Shangqiu City, Henan Province
Zhang et al. [51]	23.8	15	5	Zhengzhou City, Henan Province
Gao et al. [52]	30.1	19.2	4	Taiyuan City, Shanxi Province
Su [53]	30.80	20.00	5	Fuxin City, Liaoning Province
Zhang et al. [54]	27.20	14.17	4	Lanzhou City, Gansu Province
Zhang et al. [11]	24.00	15.00	4	Xi’an City, Shannxi Province
Jiang et al. [55]	26.4	15.7	4	Xi’an City, Shannxi Province
Gu et al. [56]	28.17	20.30	5	Xi’an City, Shannxi Province
Wang [29]	27.5	18.2	6	Lanzhou City, Gansu Province

Note: Available experimental details reported in the original studies are as follows: Wang [49], MDD = 1.536 g/cm^3^, OWC = 24%, 32.5 ordinary Portland cement, Ø40 × 80 mm specimens, and curing ages of 3–150 d; Zhang et al. [51], compaction coefficient = 0.95, Ø39.1 × 80 mm specimens, and curing ages of 7–180 d; Gao et al. [52], specimens prepared at controlled dry density and water content and cured in sealed plastic bags; Zhang et al. [54], compaction coefficient = 0.95, 425 ordinary Portland cement, and curing ages of 7–90 d; Zhang et al. [11], 95% MDD, 20 ± 2 °C curing, Ø50 × 50 mm specimens, and ASTM D2166; Jiang et al. [55], P.O 42.5 cement, compaction coefficients of 0.92–1.02, 20 ± 2 °C and 95% RH curing, Ø100 × 100 mm specimens, and curing ages of 7–90 d; Gu et al. [56], 42.5 P.O cement, 70.7 mm cubic specimens, room-temperature curing, and ASTM C109/C109M; Wang [29], P.O 42.5 cement, sealed curing at 20 ± 2 °C, Ø39.1 × 80 mm specimens, and curing ages of 7–60 d. Only information explicitly reported in the original studies is included.

## Data Availability

The data of this study are available from the corresponding authors upon reasonable request.
